# From pathology to network failure: a Question–Model–Outcome framework for interpreting animal models of AD-related and dementia-relevant mechanisms

**DOI:** 10.3389/fnagi.2026.1882211

**Published:** 2026-08-17

**Authors:** Umut Şimşekçi, Yıldırım Cesaretli

**Affiliations:** Department of Medical Pharmacology, Gülhane Faculty of Medicine, University of Health Sciences, Ankara, Türkiye

**Keywords:** Alzheimer's disease, animal models, biomarkers, dementia, network dysfunction, neuroinflammation, translational neuroscience, vascular cognitive impairment

## Abstract

Animal studies remain indispensable for research on Alzheimer's disease (AD), vascular cognitive impairment, and other dementia-relevant mechanisms. Their value, however, is not that they reproduce human late-life dementia in miniature. A mouse line, vascular manipulation, or mixed-pathology preparation can make one aspect of the problem experimentally visible while not capturing the long preclinical course, clinical heterogeneity, and everyday functional consequences that define the human syndrome. This review therefore treats animal models as instruments for bounded inference. Drawing on translational, mechanistic, and methodological literature, we ask which biological question is posed, which outcome can legitimately answer it, and where the claim should be drawn back. The Question–Model–Outcome framework is developed around five recurrent problems: the loose relation between lesion burden and functional severity; vascular influences on proteinopathy and network injury; stage-dependent immune and glial responses; the narrow construct validity of many behavioral assays; and the interpretation of reported improvement after intervention. We argue that translational value arises when model biology, disease stage, and outcome level are aligned. In that role, animal studies can sharpen mechanistic reasoning and discipline clinical extrapolation without being treated as generic substitutes for human AD-related dementia.

## 1 Introduction

Dementia first presents as a clinical problem: a progressive loss of cognitive abilities across multiple domains, eventually severe enough to disrupt ordinary life. Alzheimer's disease (AD) is the most common biological substrate of this syndrome and is classically associated with extracellular β-amyloid (Aβ) plaques and intracellular tau neurofibrillary tangles. Yet the clinical picture has never been fully explained by simply counting these lesions. Clinicopathological studies, particularly in advanced old age, repeatedly show that plaque and tangle burden correlates only modestly with cognitive status (Nelson et al., 2012).

*Working definitions used in this review*. To preserve that distinction, we use *dementia* to denote the clinical syndrome of multi-domain cognitive impairment that compromises everyday functioning and independence. We use *Alzheimer's disease (AD)* to denote the biological process defined by AD neuropathological change and validated biomarkers of amyloid-β and AD-related tau biology, whether or not dementia is present (Jack et al., 2018, 2024). We use *construct validity* in the practical experimental sense: the extent to which a model or assay captures the intended human construct—for example, executive dysfunction or network failure—rather than a narrow, task-bound skill.

By *network failure*, we mean the loss of stable cognition resulting from erosion of synaptic integrity and large-scale circuit organization. It may manifest experimentally as disconnection, hyperexcitability, or loss of compensatory capacity. *Circuit dysfunction* refers more specifically to measurable abnormalities in neural dynamics or connectivity, such as altered oscillations, excitation–inhibition imbalance, or disrupted functional coupling. *Synaptic loss* denotes structural and/or functional loss of synapses or synaptic efficacy and is treated here as a proximate substrate of cognitive impairment. *Connectivity* refers to structural and functional coupling across regions and serves as a systems-level readout of network integrity.

Finally, a *translational endpoint* is used here to denote an outcome that can support inference of clinically meaningful benefit: most convincingly, a trajectory of cognition or function, or a biomarker with a defensible relation to such a trajectory. We use *reserve/resilience* to refer to mechanisms by which individuals maintain cognition despite pathology or tolerate pathology with delayed clinical expression (Stern, 2012; Arenaza-Urquijo and Vemuri, 2018).

Human clinicopathological studies offer a useful caution when interpreting animal models: AD-type pathology is not a direct measure of dementia severity. Some older adults in community cohorts show intermediate or high AD neuropathological change or abnormal amyloid biomarkers yet remain below the dementia threshold (Azarpazhooh et al., 2020; Jansen et al., 2015). This mismatch is one reason why reserve, resistance, and resilience have become central to the field. It also counsels restraint in interpreting animal data. Aβ/tau burden may be essential for many experimental questions, but it does not automatically explain syndrome-level impairment. Cognitive decline becomes more plausible when progressive tissue injury has exceeded the remaining synaptic and network capacity for compensation (Stern, 2012; Arenaza-Urquijo and Vemuri, 2018).

This point applies even more forcefully to late-life dementia. Epidemiological and autopsy studies tend to find cognitive impairment associated with multiple lesion processes rather than a single process, including AD neuropathological change, vascular lesions, Lewy body disease, TDP-43/LATE pathology, and hippocampal sclerosis (Schneider et al., 2007; Kapasi et al., 2017; Boyle et al., 2013; Nelson et al., 2019). Animal studies gain greater credibility by specifying which aspect of the complex syndrome is being modeled: proteinopathy, vascular insult, inflammation, neurodegeneration, failure of homeostatic compensation, or some combination thereof, rather than letting a single pathology stand for the syndrome as a whole.

Diagnosis has shifted along a similar line. Biomarker studies in preclinical and prodromal AD show that biological changes can occur many years before functional deficits become noticeable (Sperling et al., 2011). In the 2018 research framework put forward by NIA–AA, biological diagnosis of AD is based on the AT(N) system, which includes amyloid, tau, and neurodegeneration (Jack et al., 2018). In the 2024 revision, early-stage Core 1 biomarkers for diagnosis are distinguished from later-stage Core 2 measures, which provide additional information about staging and prognosis (Jack et al., 2024). Question–Model–Outcome (Q–M–O) is not in competition with these criteria. Its focus is simply a different experimental question. Given a specific animal model, stage, and outcome measure, to what degree is the mechanistic or translational inference warranted? Under this interpretation, vascular insults, immune state, aging, synapse loss, and circuitry disruption are modifiers or outcome domains to be considered during translation, not replacements for AD core biomarkers (Hampel et al., 2021).

The value of animal models lies in their ability to simplify this challenging landscape under experimental conditions. The strength of transgenic mice and other systems lies in their ability to control timing, genotype, environment, and tissue sampling. Their limitation is that they are not designed to replicate the complexity of the clinical syndrome. Classic amyloid and tau models have taught us about soluble Aβ and synaptic dysfunction, and the temporal order of AD pathogenic processes (Ashe and Zahs, 2010). The list now includes knock-ins, late-onset risk models, multi-pathology models, and selected non-rodent systems (Wang et al., 2024; Collins and Greenfield, 2024). As the toolkit expands, the claims made about the models also need careful attention.

These limitations are not merely descriptive: they shape which translational claims are warranted. Many interventions that reduce pathology or improve performance in mice have not yielded comparable clinical benefit, reflecting gaps between model endpoints and patient-relevant outcomes, heterogeneity in human disease biology, and challenges in reproducibility and study design (Cummings et al., 2014; Scearce-Levie et al., 2020; Granzotto et al., 2024). Accordingly, the most constructive use of models is question-driven: models should be selected (and interpreted) in direct relation to the mechanism, disease stage, and outcome concept they are meant to interrogate.

*Contribution and scope of this review*. The contribution of this study is interpretive. We do not rank animal models by species, line, or apparent resemblance to human AD. We ask a more modest question: what claim can a particular pairing of model and endpoint reasonably support? The review therefore addresses five translational questions and uses the Q–M–O matrix in Table 1 as a compact guide, with additional operational detail in Supplementary Tables S1, S2. Table 2 serves only as a map of common model classes, not as a hierarchy. Reported “improvement” is addressed separately in Question 5 and Table 3, because improvement may mean symptomatic benefit, target engagement, network preservation, or a change in trajectory. We also keep formal AD biomarker and staging frameworks distinct from pragmatic vascular, synaptic, inflammatory, and network readouts; when these levels are blended too easily, the translational conclusion often outstrips the evidence. This is not a systematic review, a catalog of every model, or a meta-analysis. It is a question-first guide to interpretation, based mainly on mouse studies while drawing on other *in vivo* systems when they help define the boundary of inference.

**Table 1:** Compact Q–M–O matrix for the main text.

Question	Model contrast	Minimum outcome	Claim supported
Q1. Is pathology burden a suitable severity proxy?	Hold pathology background as constant as possible while varying synaptic/circuit stress, or compare pathology-targeting with circuit-stabilizing interventions.	Use pathology burden plus neurodegeneration as a proxy, a fixed synapse/circuit panel, and controlled multi-domain behavior.	Whether aggregate lesion counts are sufficient severity proxies or require synapse/circuit and behavioral confirmation.
Q2. AD pathology alone vs. overlapping vascular dysfunction?	Keep proteinopathy background fixed and add or rescue a measured vascular phenotype (e.g., hypoperfusion, BBB dysfunction, CAA-prone background).	Direct vascular readout plus AD substrate, regional/topographic mapping, white-matter/connectivity outcomes, and executive/attention plus memory tasks.	Whether vascular dysfunction lowers the threshold for impairment or changes the pattern of network injury without necessarily changing protein aggregation.
Q3. When does neuroinflammation drive, resolve, or modulate?	Use stage-aware necessity/sufficiency contrasts with cell-type/function specificity, immune/glial-state targeting logic, and resolution/recovery windows.	Proteinopathy context and stage label, immune/glial-state markers plus functional immune assay, species anchoring, and synapse/circuit outcomes.	Whether an immune or glial pathway is upstream, protective, unresolved, or modulatory within a defined disease stage.
Q4. Behavioral tests vs. human dementia?	Use a pre-specified battery spanning memory, executive/attention, and an ethological or ADL proxy, with explicit confound controls.	Multi-domain battery, motor/vision/anxiety controls, and at least one circuit-level endpoint.	Whether a behavioral effect is syndrome-relevant rather than task-specific or confounded.
Q5. What does “improvement” mean?	Compare pathology-targeting and symptomatic interventions within the model using a time-course design matched to the treatment class.	Target engagement, treatment-appropriate follow-up (washout only when relevant), circuit preservation, and slope/trajectory analysis.	Whether benefit is best interpreted as symptomatic, target-engaging, circuit-preserving, and/or disease-modifying.
Cross-cutting design variables	Treat sex, age, strain, APOE/TREM2 or other genetic background, and comorbidity as part of the model question rather than as generic nuisance variables.	Pre-specify stratification or interaction plan where feasible; sex-disaggregated and genotype/background-specific reporting for core pathology, vascular, immune, circuit, and behavioral outcomes.	Whether the supported claim is sex-general, genotype-specific, background-limited, or applicable to late-life mixed-pathology translation.

Expanded operational versions with confounds, pitfalls, and alignment cues are provided in Supplementary Tables S1, S2.

AD, Alzheimer's disease; ADL, activities of daily living; APOE, apolipoprotein E; BBB, blood–brain barrier; CAA, cerebral amyloid angiopathy; Q–M–O, question–model–outcome; TREM2, triggering receptor expressed on myeloid cells 2; VCID, vascular cognitive impairment and dementia.

*Model-selection note*. For proteinopathy questions, the model contrast should state whether the study uses non-physiological APP overexpression, physiological-expression knock-in or MODEL-AD-style late-onset risk models, or stereotaxic Aβ/tau seeding; each supports a different Q–M–O claim and different limits on syndrome-level interpretation.

**Table 2:** Representative animal-model classes and their best use in dementia research.

Model class	Illustrative examples	Best use	Main limitation
Amyloid transgenic	APP/PS1, 5xFAD	Aβ-centered synaptic and circuit mechanisms, early proteinopathy, and target-engagement studies	Non-physiological APP overexpression, familial mutations, and promoter/dosage artifacts limit sporadic late-life inference
Physiological-expression/late-onset risk models	App knock-in lines, humanized Aβ/tau platforms, MODEL-AD strains	Late-onset risk biology, age/sex/genetic-background modifiers, standardized phenotyping, and translatable biomarker alignment	Often slower, partial, or risk-factor-focused phenotypes; still require explicit pathology, circuit, and behavioral endpoint matching
Stereotaxic protein seeding/injection	Local Aβ oligomer/fibril infusion, tau seed injection	Local sufficiency, species/strain effects, regional vulnerability, propagation, and host-context modifier tests	Perturbation models; injection injury, dose/species selection, and acute timing limit claims about endogenous initiation or whole-syndrome dementia
Tau models	P301S, rTg4510	Tau-related network failure, spread, and neurodegeneration-linked dysfunction	Do not capture the full biology of AD; some lines also carry model-specific artifacts
Vascular/hypoperfusion	BCAS and related chronic hypoperfusion paradigms	VCID mechanisms, white-matter disconnection, and mixed-pathology cumulative-burden contrasts	BCAS can produce broad hypoperfusion; AD-specific inference requires measured proteinopathy, vascular readouts, and topographic interpretation
Inflammation models	LPS exposure, immune priming paradigms	Immune sufficiency/modulation tests and stage-dependent inflammatory effects	Low AD specificity when used alone
Aged animals	Aged wild-type mice, aged rats	Aging, reserve/resilience, frailty, and comorbidity background effects	Low pathology specificity
Non-mammalian models	Drosophila, zebrafish	Rapid genetic screening and pathway discovery	Weak syndrome-level translation to human dementia
NHP/companion animals	Non-human primates, dogs	Aging and cognition face validity, longer-horizon phenotypes, and comparative translational anchoring	Ethical constraints, high cost, and typically small sample sizes

The purpose is orienting rather than taxonomic: the same nominal class can support stronger or weaker claims depending on age, background strain, outcome selection, and whether mixed-pathology features are measured.

AD, Alzheimer's disease; BCAS, bilateral common carotid artery stenosis; LPS, lipopolysaccharide; NHP, non-human primate; VCID, vascular cognitive impairment and dementia.

**Table 3:** Outcome-to-inference guide for interpreting “improvement” in animal models.

Decision point/outcome	If yes, supports	If no, does not support	Common overclaim
Target engagement?	The intended biological substrate was affected; a mechanism claim may be considered.	Mechanism or disease-modification claims remain unsupported; behavioral change is more likely symptomatic, off-target, or assay-specific.	Treating a better task score as proof that the intended pathology or pathway changed.
Time course appropriate to intervention?	The behavioral effect was assessed in a window matched to pharmacology and mechanism.	Acute, carryover, practice, or exposure-timing effects remain plausible.	Applying washout as a universal rule or ignoring exposure/trajectory for long-acting interventions.
Synapse/circuit preservation?	The result can support network preservation or neuroprotection if aligned with behavior.	Target engagement alone does not demonstrate preserved neural system function.	Treating non-specific histology or aggregate pathology reduction as preserved circuitry.
Longitudinal trajectory/slope change?	A trajectory-modifying or disease-modifying interpretation becomes more defensible.	A single endpoint supports only limited rescue, symptomatic, or circuit-associated benefit.	Inferring disease modification from one terminal comparison.
Biomarker–behavior concordance?	Concordance strengthens the broadest supported claim.	Dissociation requires the narrower claim and a mechanistic explanation.	Cherry-picking the favorable endpoint when biomarkers, circuits, and behavior disagree.

*What this study is (and is not) claiming*. The recommendations rest primarily on three bodies of evidence: meta-research on reproducibility, bias, and power in preclinical science; well-described translational failures in which changes in pathology did not yield proportional clinical benefit; and framework papers that formalize biomarker and disease-stage logic, including AT(N) and related extensions. The proposed “minimum outcome sets” and decision rules should therefore be read as practical aids to interpretation, not as quantitatively validated thresholds. For this reason, we separate mechanism-focused claims—temporality, necessity, and sufficiency—from translation-focused claims about clinical mapping and treatment-relevant time course.

*Boundary conditions: where this framework is least informative*. Q–M–O is most useful for studies of late-life AD, vascular cognitive impairment, mixed pathology, or mechanisms that plausibly feed into those syndromes. It becomes less useful when the clinical anchor lies elsewhere. Tau-first dementias, primary non-AD tauopathies, frontotemporal syndromes centered on language or social behavior, and synucleinopathies with prominent autonomic, sleep, or motor disability require tighter re-scoping. The same restraint applies to mouse immune/glial programs without a clear human counterpart and to endpoints that animals cannot meaningfully approximate, such as independence in everyday activities, complex communication, or decades-long clinical trajectories. In such cases, the safer conclusion is a narrow mechanistic or biomarker-aligned claim rather than syndrome-level translation.

For that reason, the framework developed here places animal models back within the clinical problem they are often used to illuminate. It does not treat them as direct substitutes for human dementia. The review therefore uses a conceptual approach: we draw on the existing literature to examine recurring interpretive problems, rather than building the argument around individual experimental results.

## 2 Conceptual framework and method

### 2.1 Overview and rationale

However, the current study is a conceptual analysis rather than a traditional systematic review. The structure of the study reflects the order in which the five most common questions about the conditions required for drawing a valid inference from animals to humans are posed. These five questions are summarized in the main-text Question–Model–Outcome (Q–M–O) matrix (Table 1). The approach does not rely on collecting all relevant lines or calculating pooled effect sizes, but describes what a class of models may represent, which outcomes should accompany such interpretation, and where the inference process should be terminated.

The five questions consciously bring together topics that are usually considered separately. One question is related to the syndrome of dementia, which encompasses decline in cognition, behavior, and functioning without these changes necessarily occurring in parallel. Another concerns the biological continuum of AD, characterized by biomarkers. A third concerns the experimental limitations of living systems, including species differences, etiological engineering, and behavioral tests that assess only selected aspects of the human cognitive phenotype.

### 2.2 Source selection and synthesis strategy

Because clarity was the primary goal rather than numerical comprehensiveness, a narrative synthesis was conducted. We began with landmark and recent articles on the five modeling issues and expanded the source base through targeted database searches, citation tracking, reviewer suggestions, and citation-audit checks. Searches and metadata checks were organized around the Q–M–O domains, using *PubMed* as the main biomedical source, *OpenAlex* for broader scholarly coverage, and *Crossref* for DOI and publisher-metadata verification; *Semantic Scholar* was also queried as an additional academic graph source. Direct queries of Scopus and Web of Science were not performed because institutional/API access was unavailable, and Google Scholar was not scraped. The focus was on studies published from 2000 to March 2026, with older foundational papers retained when needed for clinicopathological correlation, synaptic or circuit dysfunction, and dementia staging. Search terms combined AD/dementia with animal models, transgenic and knock-in models, amyloid, tau, vascular cognitive impairment, mixed pathology, neuroinflammation, synaptic/circuit dysfunction, behavioral assays, translation, preclinical design, sex, APOE/TREM2, aging, biomarkers, and AT(N)-related framework terms.

Sources were selected purposively rather than exhaustively. We prioritized (i) framework papers that define AD, dementia, and biomarker logic; (ii) representative or seminal studies that clarify one of the five conceptual questions; (iii) recent reviews or meta-research on reproducibility and translational failure; and (iv) studies with clear mechanistic or endpoint relevance to the Question–Model–Outcome logic. Operationally, primary *in vivo* animal studies were retained for detailed synthesis when the model or manipulation and at least one endpoint could be mapped to a Q–M–O claim, such as direct pathology, vascular, immune/glial, synaptic/circuit/connectivity, or behavioral readouts. Human, biomarker, and review papers were included when they provided definitions, clinicopathological anchors, translational counterparts, or reproducibility/framework context. Conversely, we did not aim to catalog every animal line or model class. Narrowly descriptive phenotype papers, highly specialized model variants without clear relevance to the five interpretive problems, *in vitro*-only or method-only papers without a dementia-relevant *in vivo* endpoint, and model systems whose endpoints were not readily comparable to the review's dementia-focused translational questions were used selectively or excluded from detailed discussion. Accordingly, inclusion in this review should be read as *representative, seminal*, or *framework-setting*, not exhaustive.

Because the review was conceptual rather than systematic, these searches were not treated as a PRISMA review or meta-analysis. To improve transparency, however, we constructed a supplementary literature-selection and citation-audit matrix. The query log contains 29 Q–M–O-domain searches that returned 1,344 raw records across PubMed, OpenAlex, Crossref, and Semantic Scholar. After DOI/title merging, core-reference overlap checks, and relevance screening, 1,044 deduplicated hybrid candidates were screened for fit to the review's conceptual scope. The final audit matrix contains 320 DOI-verified records: 98 retained core references (97 journal articles and one book/reference record) that are all cited in the revised main text, and 222 related but unused records that were screened but not cited because they were narrower, redundant with stronger or more seminal sources, or outside the final interpretive scope. These counts document a citation audit and literature expansion process for a conceptual review; they should not be read as a PRISMA flow diagram or as evidence of exhaustive systematic screening. Supplementary Table S5 summarizes the source-selection logic, and Supplementary Data 1 provides the full literature-selection matrix with Q–M–O domain, model/system, endpoint focus, article type, DOI/PMID metadata, verification source, index coverage, search source, and selection rationale.

### 2.3 How the questions were selected

The five questions were chosen because they recur at the intersection of experimental convenience and clinical interpretation: lesion burden as a proxy for severity; co-pathologies, especially vascular injury, in late-life syndromes; stage- and context-dependent neuroimmune responses; behavioral assays that only partially approximate human dementia; and the ambiguous meaning of “improvement” in short-horizon experiments.

We treat these as organizing problems rather than as claims about a single disease mechanism. This framing keeps model selection hypothesis-dependent: the most useful model is not the most disease-like in general, but the one that most directly tests the causal or translational proposition at hand.

### 2.4 The Question–Model–Outcome (Q–M–O) matrix

The Q–M–O matrix highlights the logic behind model selection while avoiding dichotomous classification of models as “good” or “bad”. The question to be asked is what experimental comparison is being made and how that comparison is to be answered by a specific endpoint. Evidence needs to be collected before a conclusion can become scientifically plausible. In studies of either amyloidosis or tauopathy, for instance, simply counting plaques or tangles will never carry the entire interpretation of the results. Biomarker analogs or mechanistic surrogates have to be identified to match the biological assertion (Jack et al., 2018, 2024). The formal AD biomarker schema continues to play its diagnostic and staging function. Vascular (“V”), synaptic, inflammatory, and other markers are used here differently: not as alternatives to AD biomarkers, but as modifiers or outcome domains.

**Figure 1:**
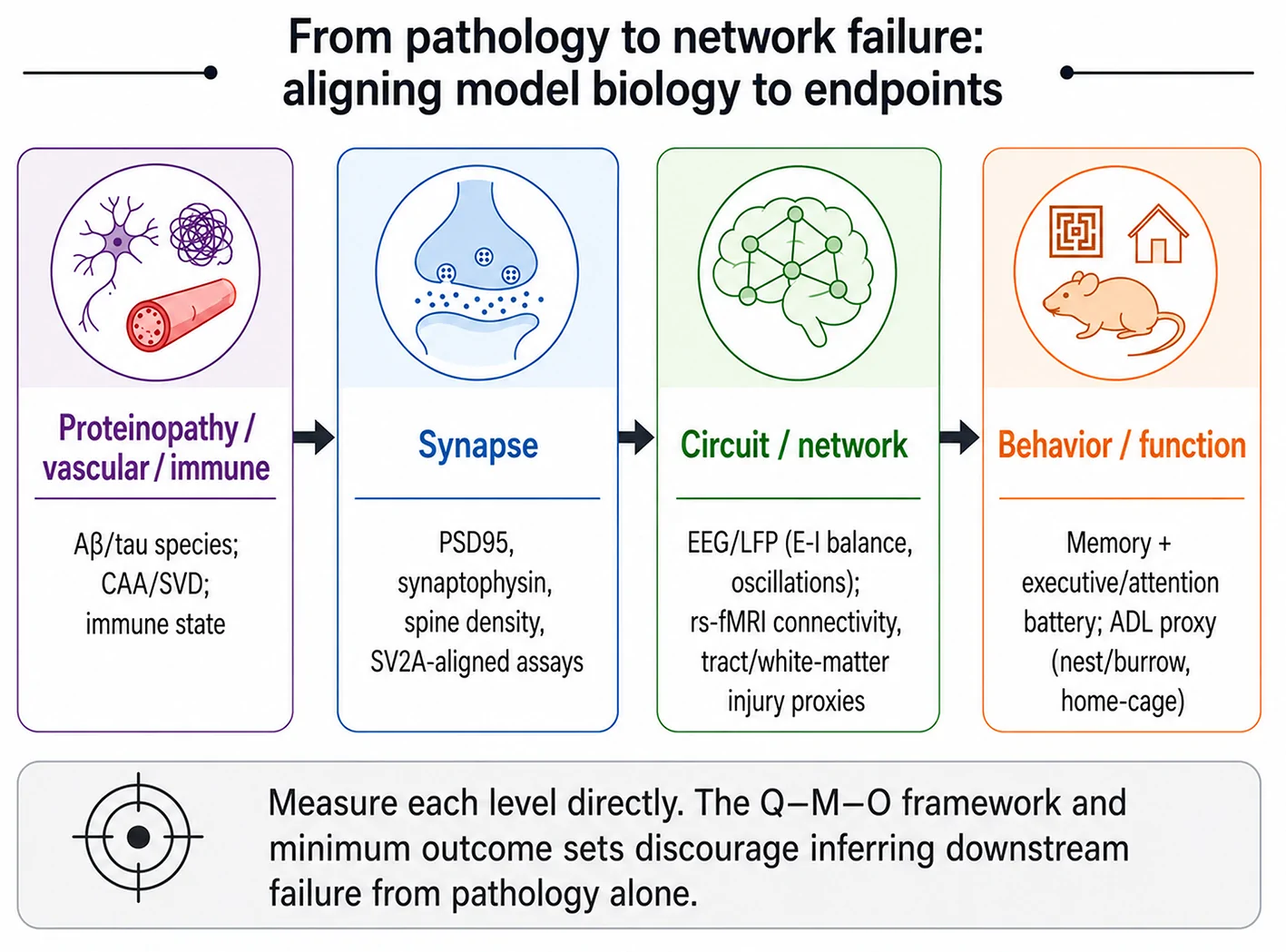
From pathology to network failure: matching model biology with endpoints. Proteinopathy, vascular injury, and immune state are shown as upstream biological pressures, whose translational meaning depends on whether downstream synaptic, circuit/network, and behavioral or functional outcomes are also measured. The figure illustrates the main Q–M–O principle: the endpoint should match the claim. ADL, activities of daily living; CAA, cerebral amyloid angiopathy; E–I, excitation–inhibition; EEG, electroencephalography; LFP, local field potential; rs-fMRI, resting-state functional magnetic resonance imaging; SVD, cerebral small vessel disease; SV2A, synaptic vesicle glycoprotein 2A.

This distinction matters when two experiments appear similar at first glance. Two amyloid-positive models may have comparable plaque loads yet differ in meaning if only one also shows synaptic loss, vascular injury, or a sustained immune response. Intervention studies face the same issue. Lowering pathology, briefly improving task performance, and stabilizing circuit activity are not interchangeable outcomes. Figure 1 traces this chain from upstream pathology to synaptic, circuit/network, and behavioral or functional readouts. The expanded matrices are provided in Supplementary Table S1 for Questions 1–3 and in Supplementary Table S2 for Questions 4–5.

### 2.5 How to apply the Q–M–O matrix

In practice, the matrix is most useful when applied before the endpoints are interpreted, rather than after a positive result has already made the conclusion tempting.

**Figure 2:**
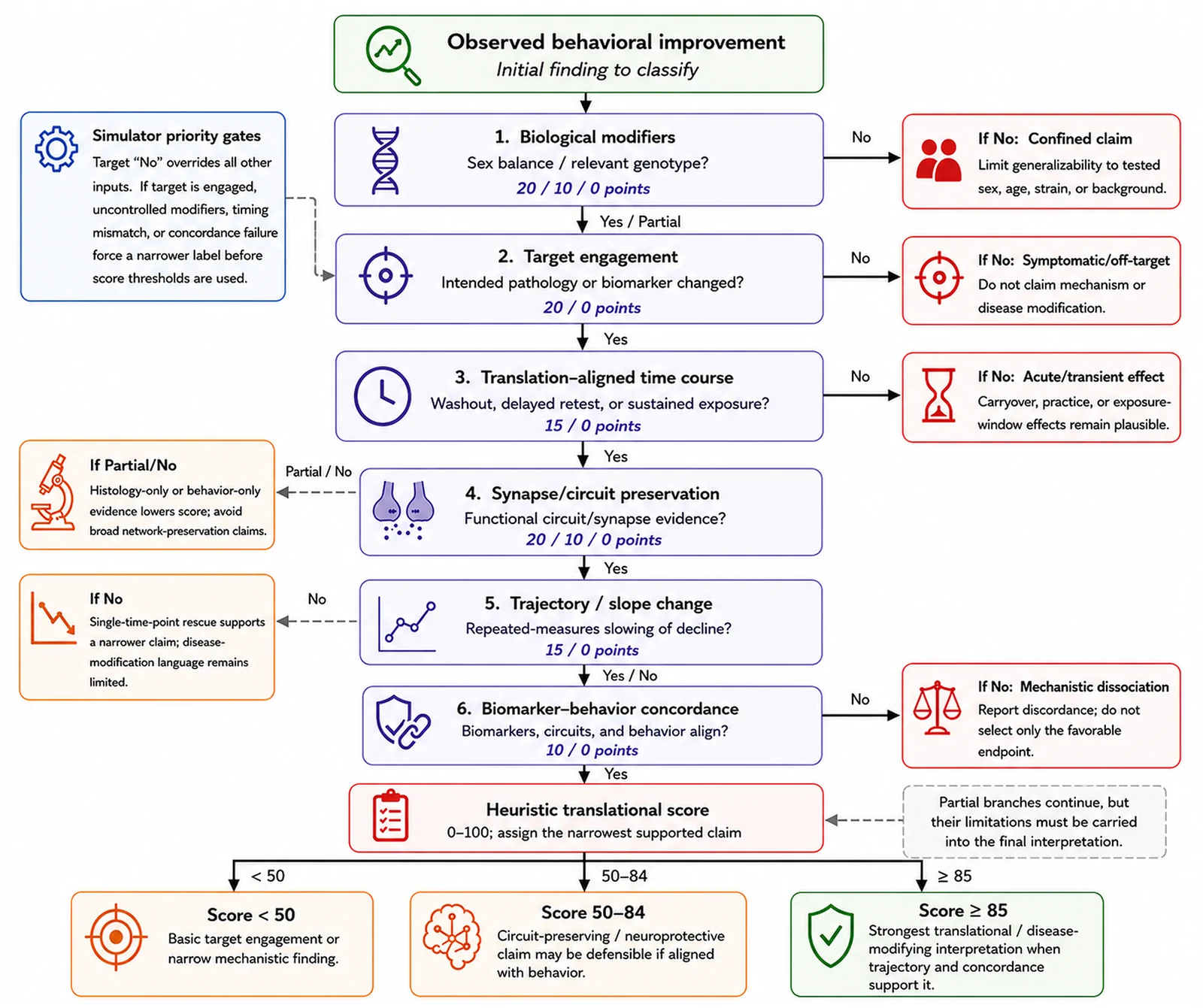
Q–M–O decision workflow for interpreting reported “improvement” in animal models. The workflow begins with a behavioral benefit and then asks whether the design addressed biological modifiers, altered the intended target, used a treatment-matched time course, preserved synapses or circuits, altered the longitudinal trajectory, and produced biomarker–behavior concordance. Negative or partial answers narrow the claim; higher heuristic scores are reserved for results that move beyond target engagement toward circuit preservation or trajectory-level disease modification.

1. *Start with the clinical setting*. The study should first state whether it is intended to inform prevention, prodromal or early intervention, or treatment at a later symptomatic stage. The model then needs to match that setting as closely as the question requires; familial-type proteinopathy, for example, should not be treated as sporadic late-life mixed dementia.
2. *Choose the main translational question*. Before selecting endpoints, the design should be tied to one primary Q–M–O domain. A secondary question can be added, but only when the design can genuinely support it rather than merely collect an additional measurement.
3. *Build the comparison around the mechanism*. The experimental contrast should isolate the biological process under investigation. Useful contrasts may compare proteinopathy alone with proteinopathy plus verified vascular dysfunction, pathway inhibition with pathway activation, or pathology-modifying treatment with circuit-stabilizing treatment.
4. *Set the core outcomes in advance*. The minimum outcome set for the chosen Q–M–O domain should be defined before analysis begins. Depending on the question, this may include diagnostic, synaptic/circuit, vascular, inflammatory, or behavioral panels. Exploratory measures can still be informative, but they should not be used later to revise the main translational claim.
5. *Name the improvement being claimed*. A reported “improvement” should be evaluated using the Question 5 decision tree (Figure 2) and decision table (Table 3). This helps prevent conflating symptomatic benefit, target engagement, circuit preservation, and possible trajectory change into a single vague positive result.

#### 2.5.1 Worked examples: applying the Q–M–O framework

The following brief examples illustrate how a positive endpoint should be converted into a bounded claim; more extensive primary-study examples are provided in Supplementary Table S6.

Q1: pathology burden as a severity proxy. Plaque reduction plus improved maze performance supports amyloid target engagement with possible behavioral benefit, but not reduced dementia-like severity unless synaptic, circuit, and behavioral endpoints move in parallel.
Q2: vascular burden as a modifier. A worse phenotype after chronic hypoperfusion indicates added vascular burden, but an independent or threshold-lowering vascular claim requires direct vascular phenotyping, appropriate single- and combined-insult arms, and, ideally, vascular-rescue evidence for circuit and behavioral outcomes.
Q3: immune/glial-state causality. Improvement after TREM2, complement, inflammasome, astrocyte, or microglial manipulation supports only a stage-, species-, and function-specific immune/glial-state effect unless temporality, necessity or sufficiency, resolution, and synapse/circuit endpoints are shown.
Q4: behavioral construct validity. A single maze improvement is task-level evidence; broader dementia-relevant claims require confound controls, a pre-specified multi-domain battery, and convergence with synapse/circuit outcomes.
Q5: interpreting improvement. A terminal behavioral benefit is not, by default, disease modification; the claim depends on target engagement, treatment-appropriate timing, synapse/circuit preservation, biomarker–behavior concordance, and repeated measures demonstrating a change in trajectory.

The sections that follow use the same basic rhythm: first, the rationale for the question; then, the designs and endpoints that can support it; and finally, the interpretive traps that most often lead to overclaiming.

**Figure 3:**
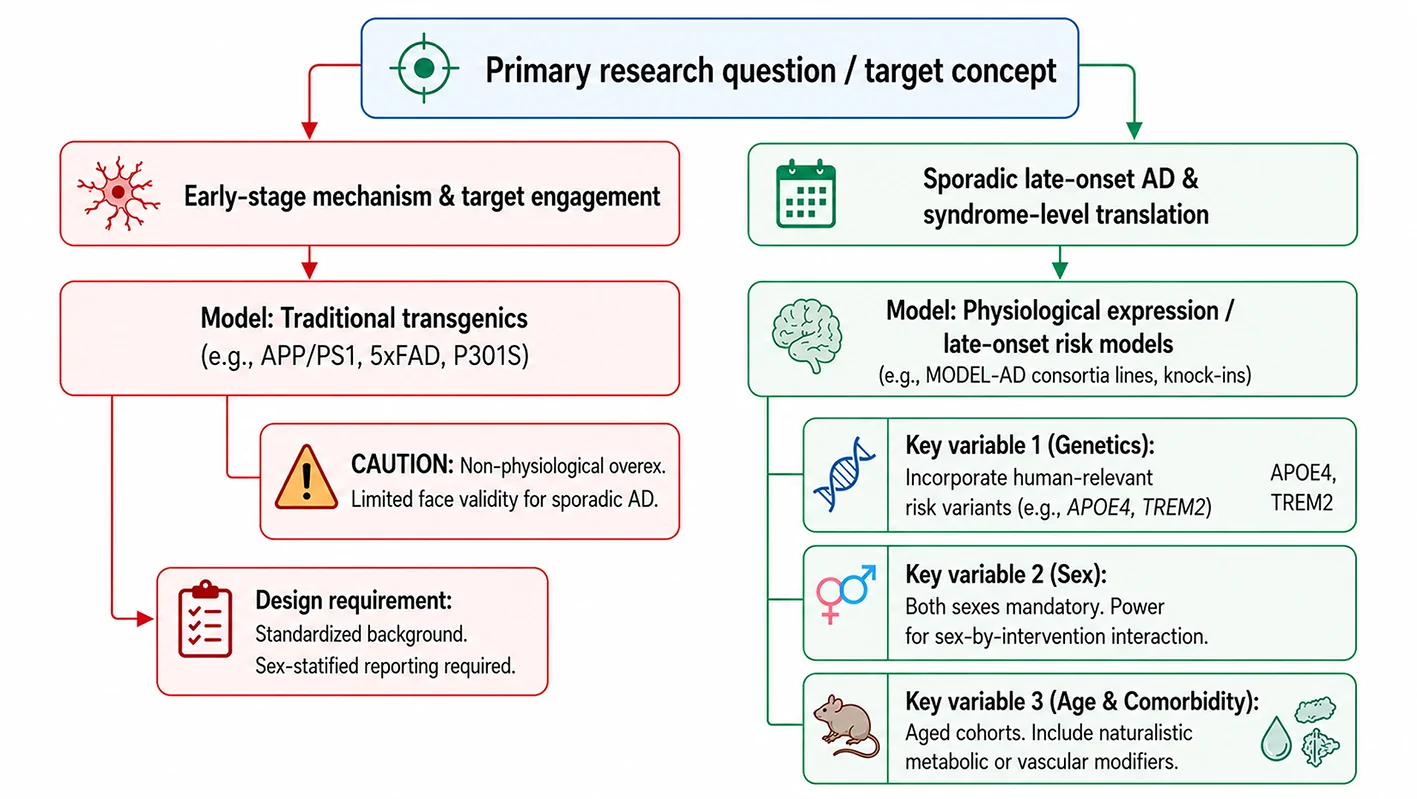
Question-first model selection for mechanism testing and late-onset translational claims. Traditional transgenic models can be useful for early mechanistic and target-engagement work, despite overexpression and limited face validity in sporadic AD. Claims about sporadic late-onset or syndrome-level translation usually require physiological expression, knock-in, late-onset risk, aged, or comorbidity-aware designs. Genetics, sex, age, and comorbidity are treated as design features that set the claim boundary.

Table 2 offers a practical map of the model landscape. The task is not to compare the models but to define the domain of applicability for each class and to mark where the model's translational potential begins to decline. For example, classical APP-overexpression transgenic models, such as APP/PS1 and 5xFAD, remain relevant for research questions about Aβ biology and target engagement. However, the very features that make these models useful also create limitations, namely artificial promoters, high gene dosage, and familial mutations, which preclude their use as an appropriate representation of sporadic late-onset dementia. Conversely, recent knock-in and late-onset risk models, including MODEL-AD resources, partially address this issue (Saito et al., 2014; Oblak et al., 2020; Scearce-Levie et al., 2020). Injection and seeding models serve a different purpose. They are useful when the question concerns regional vulnerability, molecular species, propagation, or host context, but they should not be presented as miniature endogenous disease onset or full dementia unless longitudinal pathology, circuit, and behavioral data support that broader interpretation (Meyer-Luehmann et al., 2006; Jean et al., 2015; Clavaguera et al., 2009). Figure 3 summarizes this question-first approach to model choice.

### 2.6 Rigor, bias, and interpretability

Translational claims are highly sensitive to study design, analytical decisions, and reporting practices. We therefore treat methodological rigor as a prerequisite for assessing whether an observed “model effect” is likely to be robust, reproducible, and informative.

*Design and analysis decisions*. Core recommendations for improving the predictive value of preclinical work include transparent reporting (ideally, prospective implementation) of randomization, blinding, sample-size justification, and explicit handling of attrition and exclusions (Landis et al., 2012). Underpowered studies are a particular concern because they both reduce the chance of detecting true effects and increase the probability that statistically significant results reflect inflated effect sizes or false positives (Button et al., 2013; Ioannidis, 2005).

*Systematic bias and selective reporting*. Animal experiments are vulnerable to biases such as inadequate allocation concealment, lack of blinding, and selective outcome reporting. These biases can systematically inflate apparent efficacy and complicate cross-study comparisons. Structured appraisal tools (e.g., the SYRCLE risk-of-bias instrument) make these risks explicit and comparable across domains (Hooijmans et al., 2014). Relatedly, publication bias can distort the evidence base when negative or null findings are less likely to appear in the literature; meta-research in animal studies has documented patterns consistent with such distortion (Sena et al., 2010).

*Reporting standards and study planning*. ARRIVE 2.0 helps by making the experimental unit, animals, interventions, outcomes, and statistical choices that shape interpretation clear to the reader (Percie du Sert et al., 2020). PREPARE adds a planning layer before the experiment begins, covering housing, welfare, operational definitions, and other validity threats that can otherwise remain invisible (Smith et al., 2018). For the present framework, these standards are not administrative details; they determine whether a model–outcome claim can be reproduced and bounded.

For that reason, the framework repeatedly returns to question-specific endpoints, convergence across modalities, and explicit statements about what each model can and cannot be expected to represent.

*Sex, heterogeneity, and genetic background in Q–M–O use*. Sex needs to be part of the design logic in AD and dementia experiments. It should not be treated as background variation to be averaged out. In human studies, sex-related differences have been reported in epidemiology, biomarker changes, immune biology, and clinical course (Nebel et al., 2018). When feasible, studies should include both sexes and pre-specify sex-stratified or sex-by-intervention analyses for the core outcomes. A mouse experiment using only men can still be informative, but the claim it supports is narrower. It may miss biology that is more apparent in women, obscure a sex-by-treatment interaction, or make a response appear more uniform than it would in a mixed design. Pooling men and women without testing for sex effects introduces a risk: a response present mainly in one sex may be diluted in the combined estimate.

Sex also affects how endpoints should be interpreted. A behavioral difference should not be interpreted as dementia-relevant until basic performance factors have been considered within each sex, including motor ability, sensory function, anxiety, motivation, frailty, and survival. This is especially important in aged animals, where the same task score can reflect different mixtures of cognitive and non-cognitive performance. Synaptic, vascular, and inflammatory measures can also differ in timing or magnitude. A single sampling point, or a design restricted to one sex, can therefore make a pathway appear absent when it has simply been measured at the wrong time or in the wrong subgroup.

Genetic background also shapes interpretation. APOE4 is more than a risk label; it can influence amyloid handling, tau-related injury, lipid biology, vascular function, synaptic vulnerability, and inflammatory tone, and some of these effects may vary by sex (Altmann et al., 2014; Yamazaki et al., 2019). TREM2 and other innate-immune variants shift the emphasis toward microglial phenotype, plaque handling, resolution biology, and stage-dependent toxicity (Sims et al., 2017; Jay et al., 2017; Krasemann et al., 2017). For that reason, an APP or tau line on a standard background does not ask the same question as a humanized APOE4 model, a TREM2-variant model, or a sex-by-genotype design. In Q–M–O terms, genotype should be identified either as part of the mechanism being modeled or as a boundary on how far the claim can be generalized.

An operational version of these decision rules is provided in Supplementary Table S3.

*Translational interpretation principles*. Across sections, we interpret preclinical “success” through three linked claims. First, target engagement is not the same as clinical benefit: it supports a mechanistic inference but does not, by itself, demonstrate improved function. Second, dementia-relevant impairment is treated as most proximate to *synapse/circuit integrity*—a network-first view—rather than to aggregate lesion counts alone; therefore, multi-level endpoints are needed before pathology or task scores are allowed to stand for the whole syndrome. Third, disease-modification claims are most defensible when they show a time-course or trajectory change that aligns with the treatment class, rather than a short-horizon “rescue” at a single time point. Washout is useful for some short-acting agents, whereas continuous or long-acting treatments may be better judged by sustained target engagement and slowed decline during treatment. The later sections build on these principles rather than repeating the entire argument.

## 3 Question 1: When does pathological burden fail as a severity proxy?

Lesion burden and clinical state often go together, but not in a simple one-to-one manner. At the population level, AD neuropathology is associated with dementia; at the level of the individual brain, plaque and tangle burden may still provide an incomplete account of cognitive status in late life (Azarpazhooh et al., 2020; Nelson et al., 2012). Counts are frequently used as a stand-in for severity, the validity of a model, or the efficacy of a treatment. Using that shorthand is fine if the question at hand is the nature of the lesions. Using it becomes dangerous if the statement concerns cognition, function, or preservation of neural circuitry.

The problem, then, is not whether pathology is important; clearly it is. The problem is what kind of inference histopathology is supposed to support. If the issue is cognitive decline or behavioral impairment, the same measure of pathology requires the same downstream information to be relevant: synapses, circuits, and behavior. The real question is not which, between pathology and function, is the “true” signal; it is which inference is supported by the particular measure of pathology and what further information needs to be available for that inference to be relevant.

*Lesion burden: necessary but not sufficient*. Lesion-centric approaches are right to focus on the regional extent and density of plaques or tangles. Braak staging is still useful because it captures the pattern of spread of tau pathology (Braak and Braak, 1991). But aggregate counts are only part of the tissue story. The AD brain may also include loss of synapses, neuritic degeneration, gliosis, vascular amyloid, and many other changes that do not necessarily covary (Serrano-Pozo et al., 2011).

The conceptual point is therefore not that lesion burden is unimportant. Rather, the same apparent burden can have very different clinical consequences across individuals or models. Some brains tolerate substantial pathology, whereas others show marked impairment at lower apparent burden. This pattern is consistent with reserve/resilience, the modifying effects of co-pathologies (Question 2), and the possibility that aggregate Aβ/tau burden is not, by itself, the central causal determinant of dementia-level impairment.

*When are lesion counts a useful surrogate?* Plaque and tangle measures are most useful when interpreted as stage- and region-specific biology, not as a direct severity score. They can support a mechanistic claim when paired with measures of soluble species, neurodegeneration markers, synaptic or circuit endpoints, and behavior sampled in the same animals. They are less persuasive when a single aggregate burden is used to stand in for function. Soluble Aβ oligomers, tau-related species, and circuit-level abnormalities should not be treated as secondary details in this interpretation. In many cases, they are closer to the functional deficit than total plaque area. Experimental work shows that soluble Aβ species can disrupt synaptic plasticity and memory even when plaque counts alone do not explain the phenotype (Lue et al., 1999; Shankar et al., 2008). Lesion burden is therefore an entry point for interpretation, not the final measure of disease relevance.

*Functional breakdown: synapses and networks as proximate determinants*. For animal-model severity claims, the decisive question is not only whether pathology is present but whether it has reached the neural systems that support cognition. Pathology becomes more directly relevant to dementia when it is tied to synaptic integrity, connectivity, and neuronal function. Classic quantitative human studies support this view: synapse loss in the neocortex and hippocampus correlates more closely with cognitive impairment than many gross lesion measures, making synaptic integrity a more proximal correlate of clinical status (DeKosky and Scheff, 1990; Terry et al., 1991).

At the mechanistic level, soluble Aβ and tau species can exert synaptotoxic effects and alter circuit activity, often through excitation–inhibition imbalance and network hyperactivity. These changes provide a functional link between molecular pathology and the failure of neural systems (Palop and Mucke, 2010; Tzioras et al., 2023). *In vivo* studies also show that tau pathology can impair circuit function independently of concurrent amyloid burden, arguing against aggregate load alone as a proxy for functional outcome (Busche et al., 2019).

*What counts as “network failure” in vivo?* To avoid using “network” only as a metaphor, studies should define the failure mode they intend to measure. Useful circuit-level readouts include: (i) excitation–inhibition imbalance or hypersynchronous activity, such as EEG/LFP signatures, interictal spikes, or elevated population firing (Palop et al., 2007); (ii) oscillatory measures, including theta–gamma coupling and gamma power/coherence, that index circuit organization and interneuron integrity, especially when rhythms can be experimentally modulated (Iaccarino et al., 2016); (iii) large-scale connectivity measures, such as resting-state fMRI connectivity in mouse models, that can be related to human network findings (Shah et al., 2013); and (iv) engram- or cell-assembly-level readouts, including tagging or reactivation of memory ensembles, when the claim concerns failure of the memory trace rather than task performance alone (Roy et al., 2016).

*Network dysfunction can precede dementia*. If pathology manifests clinically as network failure, network measures can become abnormal before dementia is diagnosed. Human imaging and electrophysiological studies show altered connectivity, oscillatory activity, and other network-level changes in biomarker-positive or prodromal states (Hedden et al., 2009; Sperling et al., 2011). Animal studies are useful here only when they span the same levels: pathology in defined regions, synaptic or circuit readouts, and behavior sampled with sufficient temporal resolution to determine whether network disruption precedes, tracks, or follows impairment.

*Implications for animal models and endpoints*. Many transgenic mouse lines develop prominent plaque pathology, but this pathology is not always accompanied by marked neuronal loss or strong cognitive impairment. Histological findings should therefore not be read as a stand-alone measure of disease severity or treatment response. In experimental studies, plaque or lesion burden should be evaluated alongside functional readouts, including synaptic density, electrophysiological changes, and measures of circuit connectivity. When possible, these variables should also be followed over time. This endpoint structure makes it easier to determine whether the observed impairment is more closely related to network-level dysfunction than to the absolute amount of lesion pathology.

For Question 1, this favors studies that report pathology and network-level outcomes together, use stage-aware longitudinal sampling when feasible, and examine threshold effects, such as a sharp behavioral decline after synaptic or circuit measures fall below a critical threshold. Synaptic biomarker analogs can strengthen this mapping only where they are technically available, and cross-species interpretation is explicitly justified rather than assumed (Lista et al., 2024).

### 3.1 Minimum recommended outcomes (Question 1)

To keep Question 1 interpretable across studies, we recommend a small, fixed core set rather than a flexible menu. At minimum, studies should report *pathology* (Aβ and/or tau burden, including load and regional distribution) together with a *neurodegeneration proxy* (e.g., neuron loss or cortical/hippocampal atrophy where feasible). Furthermore, to reduce selective emphasis on whichever systems readout “worked,” we recommend a *minimal network panel* that explicitly samples *all three layers* at least once per study: a synapse-level assay (e.g., synaptophysin/PSD95, dendritic spine density, or SV2A-aligned measures), a physiology/oscillations readout (e.g., LTP/LTD, excitation–inhibition balance or hyperexcitability in EEG/LFP, and/or rhythm organization such as theta–gamma coupling) (Palop et al., 2007; Palop and Mucke, 2010), and a connectivity-level measure (e.g., resting-state functional connectivity, evoked network responses, or structural/tract proxies, particularly when the claim involves disconnection) (Shah et al., 2013).

Behavioral phenotyping must be commensurate with the question being asked, but should not be based on a single task. Typically, a concise test battery comprising at least two cognitive domains is preferable, particularly when information about motor abilities, visual abilities, and anxiety is assessed concomitantly.

In this scenario, the lesion load measure remains valuable for staging and assessing target engagement. It is the more translational approach if the lesion profile is assessed in the context of synaptic or circuit dysfunction, deficits across more than one behavioral domain, and a transparent account of the main experimental confounders.

## 4 Question 2: How does vascular dysfunction modify proteinopathy and late-life translation?

Late-life dementia rarely behaves as a single-lesion disorder. Vascular brain injury, particularly cerebral small vessel disease (SVD), commonly occurs along with AD neuropathology and may reduce the threshold of AD-type lesions required for cognitive symptoms to emerge (Schneider et al., 2007; Kapasi et al., 2017; Iadecola et al., 2019). From a translational perspective, the implication is practical: if an experiment introduces a vascular manipulation into a model system, that manipulation should be identified as an explicit vascular phenotype—for example, hypoperfusion, barrier leakage, white-matter injury, impaired clearance, or another vascular abnormality—and evaluated for how it changes the relation between AD-related pathology and functional impairment.

Vascular injury is not limited to clinically apparent stroke. VCID frameworks include cerebrovascular abnormalities that can accumulate silently before cognitive deficits are recognized (Wardlaw et al., 2013; Gorelick et al., 2011; Iadecola et al., 2019). A study should therefore specify the type of vascular injury modeled. Hypoperfusion, blood–brain barrier (BBB) disruption, cerebral amyloid angiopathy (CAA), microbleeds, and impaired paravascular clearance are distinct insults that can interact with amyloid or tau biology through different mechanisms.

*Disconnection phenotypes related to SVD*. SVD-like pathological features, including white-matter abnormalities, lacunes, and microinfarcts, can weaken long-range connectivity and contribute to behavioral dysfunction even when amyloid or tau burden is not the dominant driver (Dichgans and Leys, 2017; Gorelick et al., 2011).

*Hemodynamic responses and neurovascular coupling*. Reduced cerebral blood flow and weakened hemodynamic responses can deprive active neural tissue of metabolic substrates. Over time, this may stress synapses and networks, impairing connectivity without producing a large, visible infarct (Iadecola et al., 2019).

*BBB dysfunction*. When endothelial tight-junction integrity is compromised, plasma proteins and peripheral immune signals can enter the brain parenchyma. This can trigger glial activation, oxidative stress, and synaptotoxicity, even if aggregate amyloid or tau load does not increase in parallel (Hartz et al., 2012; Iadecola et al., 2023).

*CAA and microbleeds*. In CAA, amyloid builds up in the vessel wall and makes the vasculature more fragile. When parenchymal plaques coexist with vascular amyloid and microbleeds, cognitive outcome may reflect microhemorrhagic injury and impaired vasoreactivity as much as the total amount of Aβ (Winkler et al., 2001; Gorelick et al., 2011).

*Impaired clearance*. Impairment of perivascular or venous transport can prevent the removal of Aβ and inflammatory mediators from the brain. In such cases, impaired clearance should not be viewed solely as a consequence of pathology; it can also act as an additional factor shaping the progression of proteinopathy and inflammation (Iliff et al., 2012; Iadecola et al., 2023).

Vascular and physiological abnormalities commonly affect fronto-subcortical connections, disrupting long-range connectivity. Vascular damage should therefore be viewed as a modifier of network disruption, not merely as an independent or accompanying lesion.

*Clinical data about VCID as a modifier, not a replacement, of AD biology*. Clinical data support this modifier view. Vascular risk factors, particularly hypertension, are consistently associated with later cognitive impairment, and dementia prevention frameworks identify vascular risk management as one of the more actionable ways to reduce dementia risk (Livingston et al., 2024). In SPRINT-MIND, intensive blood pressure control reduced the incidence of mild cognitive impairment and was associated with a trend toward lower risk of probable dementia compared with standard treatment (SPRINT MIND Investigators, 2019). The lesson is not that vascular treatment replaces AD biology. Rather, vascular physiology can alter the downstream cognitive trajectory and should therefore be modeled with direct vascular, synaptic/circuit, and behavioral endpoints.

*Pathophysiological routes of interaction*. Several overlapping mechanisms can connect vascular dysfunction to “Alzheimer-like” phenotypes: chronic hypoperfusion and impaired neurovascular coupling, BBB dysfunction and inflammatory signaling, oxidative stress, and impaired perivascular clearance of Aβ with accumulation in the parenchyma and vessel walls (Dichgans and Leys, 2017; Iadecola et al., 2019; Iliff et al., 2012).

*Implications for animal-model choice: explicit vascular-mechanism tests*. Question 2 is best addressed by designs that keep the proteinopathy background constant, measure the vascular phenotype directly, and avoid assuming that the added vascular manipulation is independent. In practice, we find four templates especially interpretable.

*Amyloid/tau model vs. amyloid/tau + hypoperfusion/SVD-like stress*. A common template keeps the proteinopathy background fixed and adds chronic hypoperfusion or SVD-like stress, for example bilateral common carotid artery stenosis (BCAS). BCAS is valuable because it can produce sustained hypoperfusion and white-matter injury in mice (Shibata et al., 2004). However, the interpretation should remain cautious. Proteinopathy alone can also affect vascular function. Thus, additional manipulation of vascular function may not be entirely independent of other processes; it may exacerbate the vascular pathology already present in the amyloid- or tau-based model. BCAS leads to a relatively broad perfusion abnormality, whereas amyloid- and tau-linked vascular pathologies are likely more regionally based. In these studies, perfusion and white-matter injury need to be mapped by region to be useful. What is essential to address is whether the combination modifies the anatomy of network injury, rather than only the severity of behavioral performance (Yamada et al., 2011; Kitaguchi et al., 2009).

*Amyloid/tau model vs. amyloid/tau + BBB/endothelial dysfunction*. A second template is related to barrier and endothelial function. This template is best applied when the same proteinopathy model is studied with and without a manipulation that increases leakiness or alters endothelial tight-junction/barrier function, and when BBB integrity is assessed directly. The key aspect to test is whether BBB dysfunction acts as an amplifier of inflammation, synapse loss, or circuit instability even in the absence of increased amyloid/tau load (Iadecola et al., 2019, 2023).

*Vascular rescue without reducing protein aggregation*. The strongest test of a vascular contribution is not simply adding BCAS or another stressor to a proteinopathy model. A more decisive strategy is to rescue the vascular abnormality while leaving the protein-aggregation burden unchanged, then ask whether neurodegeneration, synaptic loss, circuit dysfunction, or behavior improves. Operationally, a proteinopathy model with quantified BBB leakage, endothelial dysfunction, impaired vascular reactivity, or hypoperfusion could be randomized to vehicle vs. an endothelial-protective, BBB-stabilizing, vascular-reactivity-restoring, or perfusion-restoring intervention without concurrent anti-amyloid or anti-tau treatment. The key test is whether the vascular readout, synaptic/circuit endpoints, and behavior improve while Aβ/tau load or soluble-species measures remain unchanged. If neurodegeneration improves despite unchanged protein aggregation, the inference is stronger than a combination-stressor design. If vascular rescue normalizes the vascular readout but does not improve circuit or behavioral outcomes, the vascular phenotype is more likely to be a marker, a parallel injury, or a stage-limited modifier rather than a necessary driver.

**Figure 4:**
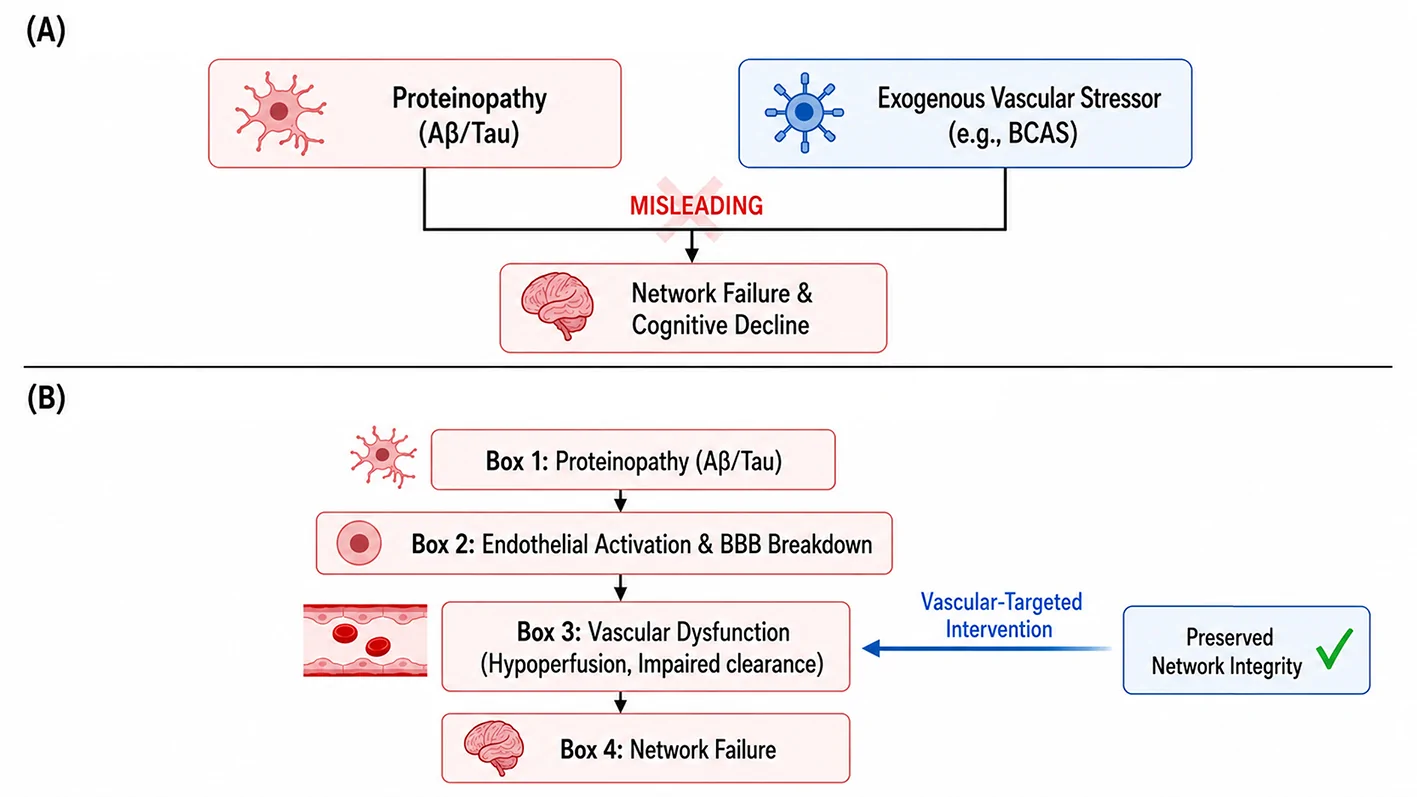
Overlapping vascular pathology and targeted rescue in Question 2. The figure cautions against a simple parallel “second hit” view, because proteinopathy can already disturb endothelium, BBB integrity, perfusion, or vascular reactivity. Broad hypoperfusion models, such as BCAS, may therefore impose overlapping vascular stress rather than a fully independent injury. The key interpretation is whether the measured vascular defect lies on the route from proteinopathy to network failure, and whether vascular rescue preserves network integrity even when Aβ/tau burden is not reduced. Here, BBB, blood–brain barrier; BCAS, bilateral common carotid artery stenosis. **(A)** The parallet “second hit” assumption (often misleading). **(B)** The overlapping pathology and targeted rescue framework (recommended).

Figure 4 summarizes why an overlapping-pathology and targeted-rescue framework is more interpretable than a simple parallel “second hit” schematic.

*Parenchymal-plaque dominant models vs. CAA/microbleed-prone models*. A third template contrasts primarily parenchymal-plaque models with CAA-heavy and/or microbleed-prone vascular-amyloid models (or induces CAA-like vascular amyloid on a controlled genetic background). This design tests whether hemorrhage-prone vascular amyloid shifts the pattern of network injury—through microhemorrhages and altered vascular reactivity—and produces a distinct cognitive phenotype compared with predominantly parenchymal amyloid, thereby clarifying which aspects of “amyloid biology” are being modeled and which translation claim is warranted (Winkler et al., 2001).

However, the key translation is typically not that “vascular injury causes AD,” but that vascular dysfunction amplifies proteinopathy, reduces reserve, and accelerates network failure.

*A compact phenotype–assay–domain map (to keep claims aligned)*. “Vascular burden” is not a single entity. To keep interpretation honest, we recommend stating the hypothesized vascular phenotype and pairing it with (i) a matched assay and (ii) an a priori prediction of the cognitive domain.

*SVD/disconnection (white-matter injury, microinfarcts)*. When the hypothesized vascular pathway is SVD-driven disconnection, the assays should directly target white-matter and microinfarct biology—for example, myelin and axonal injury markers, tract/white-matter integrity proxies, and/or microinfarct counts. The corresponding domain prediction is typically fronto-subcortical dysfunction (executive function, processing speed, and attention), consistent with SVD frameworks that emphasize cumulative, often subclinical injury to long-range connections rather than focal “stroke-like” deficits (Wardlaw et al., 2013; Dichgans and Leys, 2017).

*Chronic hypoperfusion and impaired neurovascular coupling*. When the vascular hypothesis involves reduced blood supply or diminished vascular reactivity during neural activity, perfusion and reactivity should be measured directly using flow, oxygenation, or hemodynamic-response proxies. Downstream histology alone is insufficient. The behavioral pattern may show attentional or executive slowing, while hippocampal learning effects depend on the severity, duration, and timing of the vascular challenge. Accordingly, BCAS and related chronic hypoperfusion paradigms serve as valuable titratable stress models, provided that the vascular deficit is empirically validated rather than assumed (Iadecola et al., 2019; Shibata et al., 2004).

*BBB dysfunction and endothelial activation*. Regarding BBB dysfunction and endothelial activation, the barrier phenotype requires direct quantification rather than indirect inference from downstream neuropathology. Permeability metrics, such as IgG or fibrinogen extravasation, must be co-evaluated with endothelial and tight-junction markers to confirm target engagement at the BBB. Because BBB breakdown globally amplifies neuroinflammatory signaling and synaptic stress, its functional consequences are intrinsically state-dependent and pleiotropic, rarely mapping neatly onto a discrete neuroanatomical circuit or single cognitive domain (Iadecola et al., 2019, 2023).

*CAA and microbleeds*. For models targeting CAA and microbleeds, analytical methods must distinguish vascular amyloid from parenchymal deposits. Furthermore, microbleeds and hemorrhagic micro-lesions must be systematically quantified alongside vascular amyloid burden. Given the spatial heterogeneity of CAA and consequent reactive impairments, the resulting cognitive phenotype is highly variable, potentially manifesting as executive, amnestic, or mixed deficits (Gorelick et al., 2011; Winkler et al., 2001).

*Perivascular clearance*. If impaired perivascular or glymphatic-like clearance is hypothesized to amplify proteinopathy, assays should target proxies of clearance and the resulting Aβ retention in brain and vessel walls. Domain prediction is usually indirect: clearance failure is best interpreted as an upstream amplifier of amyloid distribution and species composition, with downstream consequences for memory and network integrity that depend on where and when Aβ accumulates (Iliff et al., 2012; Bannai et al., 2019).

Within the Q–M–O framework, combination designs are useful only if the vascular component of the model is made visible rather than assumed. The strongest studies therefore specify the vascular phenotype in advance, measure it with assays suited to that phenotype, and map where vascular injury and proteinopathy overlap or diverge. They also carry the analysis downstream to synaptic and circuit endpoints, such as electrophysiology, connectivity, and synapse density, so that any behavioral change can be interpreted in relation to measured network injury rather than to the added manipulation alone.

*When is a vascular modifier essential?* A vascular modifier matters most when the target condition is late-onset, sporadic dementia, in which mixed pathology is common rather than exceptional. Adding a quantified vascular abnormality to a proteinopathy model can improve construct validity because vascular disease may reduce the amount of AD pathology the brain can tolerate before cognition fails. This does not fix the causal order in advance. Vascular dysfunction may precede, run in parallel with, intensify injury in selected regions, or develop downstream of proteinopathy. Human computational causal modeling is consistent with the idea that vascular dysregulation may occur early in a multifactorial cascade associated with AD. It may influence how Aβ and tau pathologies develop (Iturria-Medina et al., 2017). Thus, animal experiments are more revealing when they are conducted without assuming a single causal sequence in advance. Stronger designs measure regional vascular pathology, proteinopathy, and, when feasible, the effects of vascular rescue within a single experimental logic.

### 4.1 Minimum recommended outcomes for vascular phenotyping

Vascular-modifier experiments must define a specific vascular phenotype rather than treat it as a mere burden. In a hypoperfusion model, direct measurement of blood flow and vascular reactivity is required. BBB-focused models should include measurements of extravasation, along with tight junction or endothelial readouts. SVD models should estimate white-matter injury, microinfarcts, or microbleeds, while CAA-targeting models should anatomically separate vascular amyloid from parenchymal amyloid pathology.

These vascular measures should then be interpreted alongside the core AD substrates. The total burden of Aβ and tau still matters, but it does not, by itself, make a claim. When the hypothesis assumes the importance of soluble Aβ or tau species, rather than aggregation alone, those species should be included in the analysis. Sometimes the clearest experiment involves vascular rescue: do synapses, circuits, or behavior improve after correction of the vascular abnormality, despite largely unchanged aggregate pathology?

Functional outcome measures should maintain a high degree of specificity. A particular vascular lesion becomes relevant to translational research when it is linked to higher-order effects, such as changes in long-range connectivity, synaptic degeneration, or electrophysiological dysfunction. Behavioral testing should also align with the predicted vascular phenotype. Frontal/executive and fronto-subcortical tests may be as valuable as hippocampal memory tests, while sensory, motor, and frailty-related measures are also needed because vascular slowing or generally poor performance can mimic cognitive decline.

In summary, vascular dysfunction in late-life dementia models should be viewed as a modifier that lowers the threshold for proteinopathy-induced network failure. Testing this idea requires more than simply adding a vascular stressor to an AD model. It requires specific vascular phenotyping, spatial localization of injury, and rescue experiments to determine whether network preservation can be dissociated from the reduction of protein aggregates.

## 5 Question 3: When does neuroinflammation drive, resolve, or modulate pathology?

Neuroinflammation plays a role in AD biology, but the term becomes difficult to use when dissociated from immune state, time point, and tissue context. To demonstrate neuroinflammation in an animal model study, it is necessary to specify the particular glial or immune response measured, the stage of the model, and whether that response is associated with synaptic or circuit dysfunction. Some responses may support debris clearance, plaque confinement, trophic signaling, synapse preservation, or resolution in early or regulated stages. In another tissue context, especially one in which the response is prolonged, the same biology may become damaging. Examples include prolonged cytokine signaling, oxidative stress, complement-mediated synapse loss, and elevated inflammasome activity (Wyss-Coray and Rogers, 2012; Heppner et al., 2015; Leng and Edison, 2021; Buckley et al., 2014).

*Complexities of neuroimmune responses: directionality, state, and resolution*. The biological significance of a particular immune pathway changes over the course of disease. An early glial response may contribute to plaque compaction, debris removal, and tissue repair. Later, the same pathway may lose its benefits if it is prolonged, occurs in vulnerable tissue, or persists after the initial lesion has evolved. Resolution is therefore integral to the biology, not merely a technicality. An immune response returning toward homeostasis differs from an unresolved response that drives synaptic pruning, circuit instability, and cognitive decline (Heneka et al., 2015; Heppner et al., 2015; Buckley et al., 2014).

*Causality must not be inferred from association alone*. If a pathway is described as upstream, the alteration of the immune state must precede synaptic or circuit dysfunction. If the pathway is proposed to be necessary for network failure, blocking it should reduce downstream network failure. If it is sufficient, pathway activation or immune priming should cause downstream damage even in the absence of engineered proteinopathy. Resolution introduces another test: can the immune response be redirected to restore homeostasis without eliminating broad microglial function?

For this reason, each experimental description of a pathway should specify what is meant by “neuroinflammation.” The likely source cell should be identified, whether microglia, astrocytes, peripheral myeloid cells, or any combination of these. The function being tested should also be specified: phagocytosis, complement-mediated synaptic pruning, inflammasome activation, vascular–immune coupling, or another defined process. Amyloid or tau burden alone should not be used to characterize disease stage, because age, vascular or metabolic stress, and baseline synaptic integrity can determine whether an immune intervention appears protective, neutral, or harmful.

The wording should reflect the evidence. The term “driver” implies causality within a specific time frame. “Modulator” is a better choice when the pathway affects susceptibility, plaque handling, or tissue response without demonstrating a causal link to downstream network failure.

**Figure 5:**
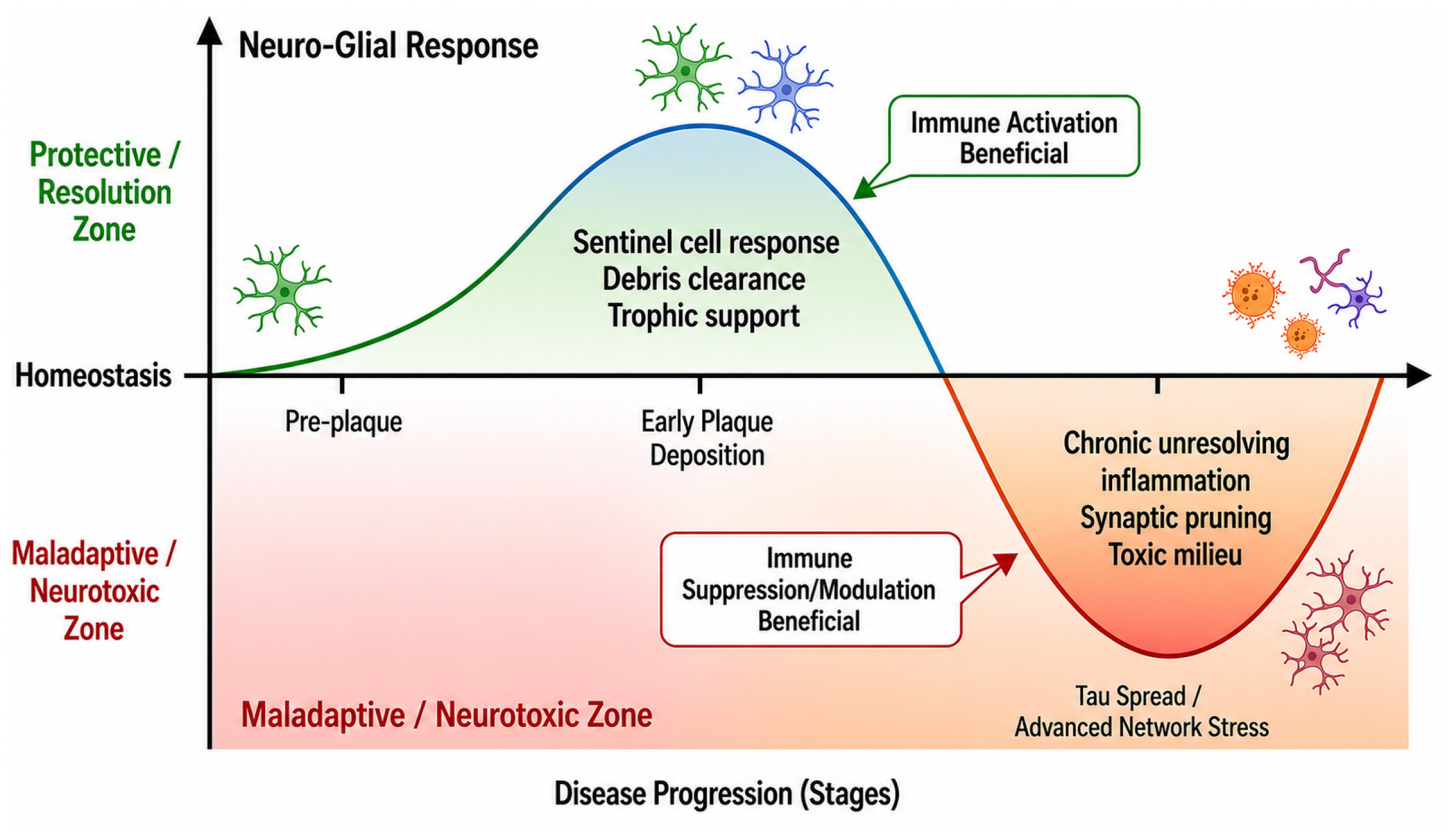
Stage-dependent neuro-glial responses during disease progression. The figure summarizes how glial and immune responses can change across disease stages. Early responses may support surveillance, debris clearance, trophic signaling, plaque containment, and resolution. With persistent proteinopathy, tau spread, or increasing network stress, the same response may shift toward unresolved inflammation, synaptic pruning, and a more toxic tissue environment. Immune activation or suppression should therefore be interpreted in relation to timing, target state, and the synaptic or circuit outcome measured.

Figure 5 is intended to demonstrate the stage-dependent interpretation of neuroglial biology: a regulated immune response might be beneficial or resolving, whereas chronic unresolved inflammation can become synaptotoxic and destabilize neuronal networks.

*The genetic data imply involvement of the innate immune system in AD*. Multiple late-onset AD risk loci are related to microglial and innate-immune biology, including TREM2-dependent pathways, phagocytosis, lipid processing, and immune modulation. Rare coding variants in immune genes imply that microglia do not only respond to injury; they also help establish conditions of vulnerability and shape the tissue response to proteinopathy (Sims et al., 2017).

*TREM2 and microglial state transitions*. The role of TREM2 illustrates why labels of “activation” and “suppression” are insufficient for describing experimental manipulations. Depending on the context, TREM2 signaling supports lipid metabolism, phagocytosis, and the confinement of damaged neurites in plaque-associated microglia. However, this effect is conditional. It depends on disease stage, aggregate load, coexisting tau pathology, and the microglial state at the time of manipulation (Jay et al., 2017; Krasemann et al., 2017). What may be beneficial in early amyloidosis can become harmful later, when chronic cytokine release, inflammasome activation, synaptic pruning, and tau-mediated degeneration dominate the local environment. Studies targeting TREM2 should therefore specify the intended phenotypic shift, the therapeutic window, and the criteria for assessing immune resolution.

*Microglial states and phenotypic transitions*. Single-cell transcriptomic analyses of AD models reveal distinct, stage-dependent disease-associated microglial signatures, with critical transition checkpoints partially regulated by TREM2 (Keren-Shaul et al., 2017).

*Species translation: mouse microglia are informative but not interchangeable with human microglia*. Mouse models are essential for causal perturbation, yet microglial programs vary across species, age, region, disease context, and experimental environment. Human microglia exhibit age- and disease-associated transcriptional features that only partially overlap with those in mouse models, and cross-species analyses reveal both conserved myeloid programs and substantial divergence in primate/human microglial signatures (Galatro et al., 2017; Masuda et al., 2019; Geirsdottir et al., 2019). Comparative single-nucleus analyses of human AD and mouse models further show that TREM2-linked and other cellular responses can diverge across species and disease contexts, limiting direct translation from DAM-like mouse states to human therapeutic targets (Zhou et al., 2020). Therefore, a mouse microglial state should not be assumed to be the human therapeutic target unless the relevant markers, direction of change, and functional interpretation are anchored in human single-cell/single-nucleus, spatial, genetic, or biomarker evidence.

*Mechanistic links between immune state and circuit failure*. Complement signaling provides a concrete example. In AD mouse models, synapses can be tagged by C1q, C3, or related complement components, making them visible to microglial complement receptor pathways. Under these conditions, immune activity is not merely a secondary byproduct of injury; it actively mediates synaptic pruning and subsequent circuit failure (Hong et al., 2016).

Inflammasome signaling provides another route from immune state to neural injury. Amyloid-β can activate the microglial NLRP3 inflammasome, and disrupting NLRP3/caspase-1 signaling in APP/PS1 models has been linked to a lower pathological burden and better cognitive performance (Heneka et al., 2013). This does not fit well with the idea that inflammasome activity is only a marker of damage. In some settings, it seems to contribute to the damaging process. Microglia-derived ASC specks may also cross-seed Aβ so that inflammasome activation may have effects beyond local cytokine release (Venegas et al., 2017). The same may be true for tau spread. Persistent microglial activation, involving exosome-linked pathways, may play a role in intercellular tau propagation rather than merely following it (Asai et al., 2015; Clavaguera et al., 2009). This does not mean that all forms of inflammation are harmful. It shows how neuroimmune activity can become pathogenic at certain stages and in certain tissue contexts.

*Models of systemic inflammation: testing immune priming, not AD etiology*. Systemic inflammation models pose an important but narrow question. An inflammatory challenge, such as repeated lipopolysaccharide (LPS) administration, can lead to cognitive impairment and increased neurodegenerative biomarkers even in the absence of transgenic amyloid overproduction (Zhao et al., 2019). These models are not intended to replicate the etiology of AD. Their value lies in testing whether peripheral immune priming, especially when it fails to resolve, can push synapses, circuits, and cognition toward a degenerative state.

*Consequences for model selection and endpoints*. When addressing Question 3, the design should first outline the immune hypothesis under test. If the claim is necessity, the immune pathway should be blocked or reduced, and the resulting synaptic, circuit, or behavioral effects should be measured. If the claim is sufficiency, immune priming or activation should induce relevant circuit injury without reliance on proteinopathy. Timing should be considered in the design because age, vascular or metabolic stress, proteinopathy, baseline synaptic/circuit condition, and the timing of intervention can all influence the outcome. Finally, resolution should be used as an endpoint: the study should ask whether the immune response returns toward a reparative or homeostatic state, not only whether inflammatory markers fall.

The endpoint set should therefore place immune measures alongside neural-system measures. Useful immune readouts include cytokines, microglial and astrocytic state markers, peripheral immune cell involvement where relevant, complement or inflammasome activation, phagocytosis/plaque containment measures, and resolution markers where feasible. These should be paired with synapse- and circuit-level outcomes, such as synaptic density, electrophysiological measures, connectivity, and longitudinal cognition. With this alignment, animal studies can distinguish a short-lived anti-inflammatory performance effect from genuine preservation of network integrity. They can also reveal the opposite problem: broad immunosuppression may remove protective glial functions along with harmful ones.

*Mini-checklist (Question 3)*. Not every useful immune study needs to satisfy all items. Descriptive, discovery, or endpoint-mapping studies remain valuable, but the strength of the claim should match the subset of criteria actually met. We therefore separate minimum requirements for strong causal or therapeutic claims from desirable strengthening criteria.

**Minimum requirements for strong causal/therapeutic claims**

- Stage window: the claim is tied to a pre-specified aging, injury, immune-state, proteinopathy, and intervention context.
- Temporality: if the claim is “driver,” the immune/glial-state shift precedes synapse/circuit dysfunction.
- Necessity or sufficiency: if the claim is causal, pathway or cell-type perturbation changes downstream circuit and behavioral phenotypes.
- Function specificity: the readout identifies phagocytosis, plaque containment, pruning, cytokine/inflammasome signaling, repair, or vascular–immune coupling rather than generic “activation.”
- Synapse/circuit endpoint: synapse/circuit measures move with or explain behavior.

Desirable strengthening criteria

- Resolution trajectory: the design distinguishes regulated/resolving inflammation from chronic unresolved inflammation.
- Target-state match: interventions such as TREM2 modulation specify the intended immune/glial state, timing, and risk of disrupting protective functions.
- Species anchoring: mouse immune/glial states are interpreted against human genetic, single-cell, spatial, or biomarker evidence where possible.
- Multi-cell context: astrocyte state, endothelial/BBB status, and peripheral immune-cell involvement are measured when invoked by the mechanism.
- Longitudinal tracking: immune/glial state, pathology, synapse/circuit injury, and behavior are sampled across a matched time course.

*Additional Question 3 examples: immune-state causality*. Because immune-state causality is particularly timing- and function-dependent, the following examples offer a deeper illustration of Question 3 rather than a separate template from the Q1–Q5 worked examples above.

*Example 1: a stage-dependent microglial causal claim*. One way to make a microglial claim convincing is to follow the model through a window in which the tissue context is changing, rather than measuring inflammation only at the endpoint. In an APP/PS1 or related amyloid model, sampling can be organized around age, early deposition, changes in immune state, and emerging synaptic or circuit abnormalities. The key point is temporal ordering: the microglial change of interest—for example, a DAM-like profile, plaque-barrier formation, or complement engagement—should precede measurable synapse loss or behavioral decline.

A causal claim about TREM2 or a related microglial pathway should not rest on correlation alone. If the pathway is manipulated, the study needs to demonstrate, in the same experimental setting, that the intended microglial phenotype changed and that synaptic or circuit integrity changed with it (Jay et al., 2017). The stage of the model is critical to this interpretation. A microglial profile that helps contain early plaques may lose that role, or even become harmful, if resolution fails. Likewise, broad microglial suppression may have different consequences depending on when it is applied (Spangenberg et al., 2019).

For this reason, glial markers should not be interpreted in isolation. When the claim concerns circuit protection or injury, synaptic density, EEG/LFP oscillations, or network connectivity should be measured directly. The point is not that microglia initiate amyloidogenesis, nor that they are uniformly protective or destructive. Rather, specific glial programs may influence the threshold at which amyloid, tau, vascular injury, or systemic inflammation begins to produce circuit toxicity.

*Isolating specific microglial functions*. A more informative design separates general microglial dependence from a defined microglial function. Pharmacological depletion and repopulation, including CSF1R inhibition, can be timed to specific disease stages and paired with pathway-specific genetic perturbations. The relevant function may be complement-mediated synaptic pruning, plaque containment, inflammasome amplification, tissue repair, or another defined process. The key question is whether altering that function affects synaptic loss, circuit instability, or cognitive decline, not merely whether the total microglial population has changed (Hong et al., 2016; Spangenberg et al., 2019).

### 5.1 Minimum recommended outcomes (Question 3)

At a minimum, we recommend anchoring immune findings in their *proteinopathy, aging, vascular/metabolic injury, and immune-state context*: report Aβ/tau trajectories when relevant (including soluble species when feasible), model age, sex, genotype/background, vascular or systemic-inflammatory stressors, intervention window, and synapse/circuit stage explicitly, because immune states and their functional meaning are strongly context-dependent.

Second, we recommend measuring *immune/glial state and resolution rather than generic “activation.”* At least one state-resolved readout should be included, such as microglial markers aligned with disease-associated programs (e.g., DAM-like signatures) (Keren-Shaul et al., 2017; Krasemann et al., 2017) and/or a targeted transcriptional panel or single-cell/single-nucleus readout that avoids treating microglia as a single homogeneous endpoint (Keren-Shaul et al., 2017; Mathys et al., 2019). Where feasible, this should be paired with a time-course readout that distinguishes transient, regulated inflammation from chronic, unresolved inflammation (for example, persistent cytokine/complement/inflammasome activity, failure to recover homeostatic markers, or impaired clearance/repair). When the hypothesis involves glial crosstalk, astrocyte activation/state markers, complement/cytokine signaling, metabolic/BBB-support readouts, and peripheral immune-cell or systemic-inflammatory measures should be included as co-readouts rather than afterthoughts.

Third, we recommend a *functional immune core panel* that includes at least one functional assay aligned with the hypothesized mechanism. Depending on the claim, this can include phagocytosis/clearance capacity (e.g., uptake assays, efferocytosis proxies, or plaque-associated microglial barrier metrics), synaptic engulfment/pruning proxies such as complement tagging and microglial engulfment of synaptic material (Hong et al., 2016), and/or inflammasome activation/readouts when that axis is implicated (Heneka et al., 2013; Venegas et al., 2017).

Fourth, we recommend adding a *species-translation check* for immune/glial claims. If a mouse study uses a DAM-like, TREM2/APOE-linked, astrocyte-reactivity, interferon/cytokine, or complement/inflammasome state as the key endpoint, the manuscript should state whether that state has a human counterpart and whether the direction of change is supported by human genetics, postmortem single-cell/single-nucleus data, spatial datasets, or biomarker evidence (Galatro et al., 2017; Masuda et al., 2019; Geirsdottir et al., 2019; Mathys et al., 2019; Zhou et al., 2020).

Finally, immune outcomes should be interpretable against *circuit/synapse integrity* (synapse density plus at least one circuit-level endpoint such as electrophysiology, connectivity, or neuronal injury markers, ideally longitudinal) and against *behavior*, preferably longitudinal cognition when feasible and with pre-specified primary outcomes to limit selective reporting.

*Evidence basis and takeaway (Question 3)*. Human genetics, perturbation studies, active-resolution biology, microglial depletion/pathway studies, and single-cell/state-mapping work support interpreting microglia, astrocytes, vascular–immune interfaces, and peripheral immune contributions as dynamic state-dependent processes; without temporality, necessity or sufficiency, resolution/recovery information, species anchoring, and synapse/circuit endpoints, inflammation outcomes should remain bounded immune-state associations.

## 6 Question 4: Do behavioral tests in animals reflect human dementia?

Rodent cognitive assays typically assess narrow domains (often hippocampal-dependent memory) under highly controlled conditions. Conversely, human dementia is characterized by multi-domain impairment and loss of functional independence. This mismatch creates two recurring risks: (i) overgeneralizing from single-task deficits to “dementia,” and (ii) overvaluing task improvement as evidence of clinically meaningful benefit.

*Construct validity is the central problem*. A single task rarely maps cleanly onto a clinical construct (e.g., “episodic memory” or “executive dysfunction”), because performance is influenced by motor capacity, vision, stress reactivity, and strategy learning. Accordingly, we recommend treating any one assay as a noisy indicator and using convergent evidence across tasks and outcome modalities (Crawley, 2007; Crawley and Paylor, 1997).

*From “one task” to batteries and domain coverage*. Dementia is a syndrome-level impairment; therefore, animal-model inference improves when behavioral assessment spans multiple domains rather than a single hippocampal task.

*A domain map: what rodents capture well vs. poorly*. Because “multi-domain” can be vague, we recommend explicitly stating which clinical domains are approximated and where construct validity is strongest, consistent with behavioral-phenotyping guidance that emphasizes domain coverage and convergent evidence over single-task claims (Crawley, 2007; Crawley and Paylor, 1997).

In practice, *spatial/recognition memory and learning* are often captured reasonably well, particularly when hippocampal-dependent assays are replicated across task formats so that a phenotype is not an artifact of a single maze, exploration strategy, or training pipeline. *Executive-function components* (cognitive flexibility, reversal learning, sustained attention) can also be modeled with relatively higher construct leverage, especially in touchscreen-based paradigms that help separate learning, attention, and impulsivity components and standardize task structure across laboratories (Oomen et al., 2013; Mar et al., 2013).

Conversely, some clinical constructs are *typically weaker targets* for direct rodent modeling and therefore carry a higher risk of over-interpretation. “Human episodic memory” in the strict autobiographical/temporal-context sense is difficult to operationalize because most rodent assays capture simpler recognition, spatial navigation, or rule learning rather than narrative- and context-rich recollection. Similarly, *language* and aspects of *complex social cognition* central to several human dementias have limited construct correspondence due to species differences in communication and social inference. Finally, *functional independence/activities of daily living (ADL)* are clinical endpoints that rodents cannot model directly; the most defensible approach is therefore to treat “function” as a proxy construct and rely on species-typical home-cage behaviors and long-horizon monitoring rather than implying direct equivalence to human independence (Crawley, 2007).

*When to include social assays (and when to omit them)*. Because social cognition is both clinically important and difficult to model, we recommend treating social tasks as *hypothesis-triggered* rather than default endpoints. Social assays are most defensible as essential outcomes when (i) the target syndrome involves prominent social or behavioral change (e.g., frontotemporal-spectrum phenotypes or interventions aimed at social-affective circuitry), (ii) the manipulation is expected to alter motivation, anxiety, or social drive, so apparent “cognitive” task changes may be secondary, or (iii) the primary behavioral endpoint is intended to approximate functional impairment beyond hippocampal memory.

Conversely, it is reasonable to omit social assays when the model question is narrowly mechanistic (e.g., synaptotoxicity or vascular disconnection) and the behavioral hypothesis is primarily memory/executive, or when the study is underpowered for a multi-domain behavioral expansion. In that case, a small core battery plus required confound controls is usually more interpretable than an underpowered broad battery (Crawley, 2007; Crawley and Paylor, 1997).

*Interpretation check: avoid mistaking social effects for cognition*. If social assays are included, report anxiety/motivation and locomotor controls in the same animals and interpret social changes as potentially independent of (or upstream of) cognitive task performance.

*Ethological “activities of daily living” (ADL) proxies and digital phenotyping*. Because human dementia is partly defined by loss of everyday function, ethological measures can add face validity and reduce over-interpretation of narrow laboratory skills. Nest building and burrowing are widely used, easy-to-quantify home-cage behaviors that can be sensitive to neurological dysfunction and disease progression (Deacon, 2006a,b; Jirkof, 2014). Furthermore, automated home-cage monitoring can provide long-horizon, low-stress behavioral readouts (sleep–wake, locomotor structure, grooming, circadian fragmentation) that are closer in spirit to “functional” outcomes than brief single-session tasks (Jhuang et al., 2010; Mingrone et al., 2020).

*A minimal operational core battery for Question 4*. For most dementia-model studies, we recommend pre-specifying a small core battery and control panel that separates cognition from confounds, and can be repeated longitudinally.

A concrete operational version of this minimum battery and control panel is provided in Supplementary Table S4.

### 6.1 Common failure modes and required controls (Question 4)

At a minimum, three common confound domains should be measured rather than assumed away. Motor performance, including gait, locomotion, and strength, is necessary to avoid mistaking slowing for cognitive impairment. Vision and other sensory functions should be assessed because maze or touchscreen failure can reflect poor cue detection rather than memory or executive dysfunction. Anxiety, arousal, and motivation also need explicit readouts, since changes in exploration, reward seeking, sickness behavior, or sedation can mimic cognitive change in standard tasks.

*When the behavioral conclusion should remain provisional*. Behavioral language should be narrowed when plausible confounds travel with the apparent cognitive endpoint: locomotor slowing, sensory loss, anxiety, weight loss, sickness behavior, or sedation. The same caution applies when the claim rests on a single task, when training or handling differs across groups, when dropout during shaping is not reported, or when criterion decisions are not blinded. A behavioral improvement is also weak evidence of relevance to dementia if no synaptic or circuit endpoint is measured, or if the timing of testing is mismatched with the intervention—for example, acute testing of a short-acting drug or no trajectory data for a long-acting treatment.

*Interpretation rule*. We recommend pre-specifying primary behavioral endpoints and explicitly reporting exclusion/attrition rules, consistent with broader reporting guidance for animal experiments (Percie du Sert et al., 2020).

#### 6.1.1 Minimum recommended outcomes (Question 4)

At a minimum, we recommend reporting a pre-specified *behavioral* set that includes one memory assay and one executive/attention assay, along with an *ADL proxy*, such as nest building/burrowing and/or a home-cage monitoring summary metric. These behavioral outcomes should be reported alongside required *control measures*—motor/locomotor screening, vision/sensation screening, and anxiety/motivation screening—to support construct-valid interpretation (Crawley, 2007).

To link behavior to mechanism, we further recommend at least one *circuit/synapse integrity* endpoint (so that behavioral change can be interpreted as neural-system change rather than a task strategy) and explicit reporting of the relevant *pathology/biology* invoked by the model (Aβ/tau, vascular lesions, and/or immune pathway engagement) so that behavioral patterns can be interpreted in relation to the intended substrate.

*Evidence basis and takeaway (Question 4)*. Behavioral-phenotyping frameworks and meta-research on bias support using pre-specified batteries, confound controls, and ADL-like proxies as conservative coverage checks rather than validated clinical surrogates; single-task improvement is most meaningful when it converges with synapse/circuit preservation.

## 7 Question 5: What does “improvement” in a model mean, and how does it translate?

In animal studies, the term “improvement” often encompasses several distinct findings. It can refer to better task performance, reduced pathology, preserved synapses or circuits, or a slower rate of decline. These findings should not be treated as equivalent. *Symptomatic improvement* indicates better performance without clear evidence that disease biology has changed. *Target engagement* indicates that the intended substrate has changed, such as soluble Aβ/tau species, plaques, tangles, vascular markers, or immune markers. *Neuroprotection or network preservation* requires evidence that synapses, neurons, white matter, or circuit function are preserved. *Slowed decline* requires repeated measures showing a change in trajectory, not merely a better score at a single time point.

These outcomes can dissociate. Symptomatic treatment can improve behavior without altering pathology. Pathology-targeting treatment can reduce plaques or tangles without restoring cognition if synaptic or circuit injury is already established. Therefore, a model result should state exactly which claim is supported: symptomatic effect, target engagement, circuit preservation, slowed decline, or a combination of these. The strongest translational claim requires alignment among the intended target, circuit-level outcomes, and cognition or function.

To prevent improvement claims from becoming too broad, we use the Figure 2 workflow as a set of checks rather than a rigid scoring rule. The first check is design context: were sex balance, genotype, and other generalizability limits addressed or at least stated? The second is target engagement: did the intended substrate actually change? The third is timing. A short-acting compound may require washout or delayed retesting to separate transient performance effects from persistent benefit, whereas antibodies, gene therapies, long-acting injectables, and continuous treatments are better evaluated during the relevant exposure period, with stable target engagement, dose-interval logic, or a maintained difference in the rate of decline. The fourth check asks whether synapses or circuits were preserved, not only whether behavior improved. The fifth asks whether the evidence comes from a slope or a single endpoint. The last asks whether biomarkers, circuits, and behavior tell the same story. The optional Q–M–O Decision Tree Simulator was built by the authors as a web-based companion to this reasoning process; it guides users through biological modifiers, target engagement, treatment-matched timing, synapse/circuit preservation, trajectory change, and biomarker–behavior concordance. The live tool is available at https://umutsimsekci.github.io/qmo-simulator/, with source code at https://github.com/umutsimsekci/qmo-simulator. Its 0–100 output and labels are intended as an over-claiming prompt, not as a validated prognostic, diagnostic, or regulatory measure. An implementation summary of the simulator is provided in Supplementary Table S7.

*A compact summary table*. Table 3 summarizes the decision points and the corresponding interpretation targets.

For long-acting treatments, a washout phase is not automatically the most informative design. Antibodies, gene therapies, slow-release formulations, and continuous treatments are meant to act during sustained biological exposure. In these cases, stopping treatment solely to create a washout period may address a different question than the one that is clinically relevant.

The timing rule should therefore align with the treatment mechanism. For antibodies, the key evidence may be sustained target engagement. For gene therapies, it may be durable transcriptional or cellular modulation. For slow-release formulations, it may be preservation of the effect throughout the dosing interval. A withdrawal phase can still be useful, but it should be justified by a safety question, a clinical-use scenario, or a specific mechanistic hypothesis. It should not be used as a default requirement for claiming disease modification.

In AD trials, efficacy usually means slowing decline rather than restoring baseline function. Preclinical studies supporting a disease-modifying claim should therefore include sufficient repeated measurements to distinguish a true change in trajectory from short-term fluctuations. A baseline and two follow-up points may suffice for a simple slope estimate, but denser sampling is needed when the phenotype changes rapidly, non-linearly, or near a threshold. Repeated testing also introduces its own problems. Alternate task versions, counterbalanced test order, and analysis of practice effects may be needed. In aged or advanced-pathology cohorts, attrition should also be treated as part of the phenotype, because death and dropout may reflect disease severity.

A therapeutic interpretation is strongest when several lines of evidence converge: the intended molecular target changes, synaptic or circuit integrity is preserved, behavioral findings are not explained by motor or sensory confounds, and the follow-up window aligns with the pharmacology. Recent anti-amyloid antibody trials are particularly relevant here because they illustrate why the nature of the benefit must be specified carefully. Lecanemab and donanemab reduce amyloid, but their main clinical signal is slowing of cognitive decline, not recovery of baseline function (van Dyck et al., 2023; Sims et al., 2023). Preclinical studies should exercise the same caution. They need to state what changed biologically, when it was measured, and how far the result can be carried toward a translational claim.

*Summary and minimum requirements (Question 5)*. In Question 5, “improvement” is not a single outcome. It may refer to symptomatic benefit, biochemical target engagement, structural preservation, network maintenance, and/or slowing disease progression. These meanings should be kept separate. A dissociation between behavioral and biochemical, structural, or circuit effects does not necessarily mean the study has failed. It may still be mechanistically useful. However, in most cases, it should narrow the clinical claim rather than broaden it.

## 8 Conclusion

The Q–M–O framework has a specific and deliberately limited purpose. It is not designed to rank animal models in general. Rather, it asks what a given model can demonstrate when assessed at a defined disease stage and against a defined endpoint. A model may therefore be highly informative for a specific mechanistic question, while offering much weaker support for broader claims about late-life dementia.

Throughout this review, dementia-relevant impairment has been interpreted primarily as a failure at the synaptic and network levels. This emphasis does not diminish the importance of Aβ or tau, both of which remain central to AD biology and to many experimental questions. However, proteinopathy alone does not fully account for the clinical syndrome. Vascular injury, immune status, aging, genetic background, biological sex, and the loss of compensatory capacity may all influence when pathology becomes clinically apparent and how it progresses toward cognitive and functional impairment.

This distinction is particularly important when interpreting evidence from controlled amyloid or tau models. Such models can provide strong evidence for target engagement, pathway isolation, or a defined molecular mechanism. They provide weaker support for claims about sporadic late-onset dementia when mixed pathology and age-related modifiers are not represented. In this sense, the main limitation often lies not in the model itself, but in the inference drawn from it.

Similar caution is required when interpreting treatment effects. An acute behavioral improvement may indicate symptomatic benefit, whereas a biomarker shift may demonstrate target engagement. Neither outcome, on its own, shows that synapses or circuits have been preserved; nor is either sufficient to establish disease modification. Interpretation should therefore remain aligned with the measured outcome, the timing of assessment, and the duration of follow-up.

In our view, a narrow but well-supported conclusion is more valuable than a broad translational claim that depends on assumptions not directly tested. The purpose of the Q–M–O framework is to clarify this boundary.

## 9 Future directions

Building on this conclusion, future work will require complementary model systems, each tailored to a specific biological or translational question. No single experimental system is likely to capture the full biology and time course of late-life dementia. Rodent models will remain essential because they enable genetic manipulation, longitudinal studies, and experimental interventions that cannot be performed in humans. Their main strength, however, is experimental controllability, not whole-syndrome resemblance (Wang et al., 2024; Collins and Greenfield, 2024).

Different platforms should therefore be used for different claims. Rats may support richer behavioral phenotyping. *Danio rerio* and *Drosophila* can accelerate genetic and early pathway screening. Non-human primates may be useful for selected circuit-level questions, although ethical, economic, and sample-size constraints are substantial. Companion-animal cohorts and humanized *in vitro* systems may provide complementary information on aging, environmental exposures, and cell-type-specific biology, while introducing their own limits in experimental control and temporal modeling (de Sousa et al., 2023).

Mixed-pathology designs deserve greater attention when the intended inference concerns sporadic late-onset dementia. Single-lesion models remain valuable for isolating mechanisms, but they cannot by themselves capture the frequent co-occurrence of amyloid, tau, vascular injury, inflammation, frailty, and risk-factor biology in older adults (Schneider et al., 2007; Kapasi et al., 2017; Livingston et al., 2024). Incorporating age, biological sex, human genetic risk, vascular or metabolic stress, and longitudinal staging may be more informative for late-life questions than relying only on early-onset, single-hit transgenic paradigms. Such complexity should be added when the hypothesis requires it, not as a default.

Network-level preservation needs synaptic or circuit-level evidence in addition to reduction in aggregate pathology (Terry et al., 1991; Tzioras et al., 2023). Cognitive or functional claims should not be based on performance in a single favorable behavioral task. Behavioral experiments need motor, sensory, anxiety, motivational, and frailty-related controls to prevent changes in non-cognitive performance from being interpreted as cognitive gain (Crawley, 2007; Oomen et al., 2013; Deacon, 2006a,b). Negative or discordant results should also be reported clearly, because they can help identify the point at which the model no longer supports the original hypothesis.

Animal endpoints should be cautiously mapped to human biomarkers. Human-aligned endpoints are relevant only insofar as the corresponding animal assay is validated for that platform and interpreted within its biological limits (Jack et al., 2018, 2024; Hampel et al., 2021). Fluid and imaging biomarkers, including plasma *p*-tau panels and synaptic neuroimaging, can improve comparison with human studies, but they do not make animal endpoints equivalent to clinical outcomes (Barthélemy et al., 2024; Palmqvist et al., 2025). Table 4 presents these mappings and differentiates the formal AT(N) framework from vascular, synaptic, inflammatory, and network measures used as complementary translational markers.

**Table 4:** A compact translational mapping legend: animal endpoints, human counterparts, and what they can (and cannot) justify.

Preclinical endpoint	Closest human counterpart	Interpretation note (what it does/does not justify)
Amyloid/tau pathology change, including soluble species.	Formal AT(N) A/T biomarkers: amyloid/tau PET; CSF/plasma amyloid and *p*-tau panels.	Shows that the intended AD substrate changed; by itself, it does not show better cognition or daily function (Question 5).
Vascular phenotype readouts: perfusion/reactivity, BBB leakage, WM injury, microinfarcts/microbleeds, CAA	Vascular information used in mixed-pathology/VCID framing: MRI WMH/ lacunes/ microbleeds, vascular reactivity, BBB markers	Confirms that a vascular component is actually present in Question 2; without it, vascular-modifier or cumulative-burden language is speculative.
Synapse/circuit preservation: SV2A-aligned assays, electrophysiology, connectivity	Injury biomarkers plus non-AT(N) synaptic/network adjuncts where validated and available, such as SV2A PET, EEG/ MEG, or connectivity	Places the result closer to cognitive-system biology than aggregate pathology alone; it supports a network-preservation claim rather than a purely histological one.
Behavioral battery scores	Human cognitive domains and function: memory, executive/attention, processing speed, and activities of daily living	Extends the behavioral construct beyond a single task, while remaining limited by species and assay design; motor/sensory controls and circuit evidence are still needed (Question 4).
Single-time-point “rescue”	Clinical cross-sectional score difference	May reflect symptomatic benefit, practice effects, or short-term rescue; disease-modification claims require treatment-matched follow-up and slope evidence.
Repeated longitudinal measures under relevant exposure	Slower decline or maintained function	Offers the closest animal-level parallel to a clinically meaningful trajectory, provided attrition, timing, and endpoints are planned before analysis.

*Terminology note*. AT(N) refers only to the formal A/T/N biomarker framework. The vascular, synaptic, inflammatory, and network entries are practical translational additions and should not be read as standardized AT(N) categories; “closest human counterpart” indicates conceptual similarity, not validated interchangeability.

Future treatment studies should examine disease trajectories rather than rely primarily on a single terminal rescue assessment. Treatment windows should align with plausible clinical stages, and progressive phenotypes should be measured repeatedly. This is necessary to distinguish transient symptomatic improvement from sustained target engagement, circuit protection, or a genuine slowing of decline (van Dyck et al., 2023). When disease stage, species correspondence, behavioral construct validity, or treatment timing has not been tested directly, the translational claim should remain correspondingly limited.

## Data Availability

The optional companion Q–M–O Decision Tree Simulator was designed and developed by the authors and is available as a live GitHub Pages tool at https://umutsimsekci.github.io/qmo-simulator/, with source-code repository access at https://github.com/umutsimsekci/qmo-simulator. The simulator is provided as an implementation aid for the framework and is not a source of empirical data.
