# Supplementary material for: From pathology to network failure: a Question–Model–Outcome framework for interpreting animal models of AD-related and dementia-relevant mechanisms
Source: Front Aging Neurosci. 2026 Aug 17;18:1882211. doi: 10.3389/fnagi.2026.1882211 (PMC13526821; doi:10.3389/fnagi.2026.1882211)
Supplement: Supplementary file 1 [file Data_Sheet_1.pdf]

Supplementary Material

1 SUPPLEMENTARY DATA

This supplementary file expands the operational details referenced in the main manuscript. The record-level literature-selection and citation-audit matrix is provided separately as Supplementary Data 1. In keeping with the Frontiers supplementary structure, this file does not repeat the title, author list, affiliations, or correspondence information.

2 SUPPLEMENTARY TABLES AND FIGURES

The supplementary content below follows the same Frontiers-style page layout, with numbered headings and the original design preserved.

2.1 Tables

Table S1. Expanded operational Q–M–O matrix

Q	Model contrast	Minimum endpoint set	Supported claim
Q1	Proteinopathy model paired with direct circuit phenotyping or dissociation designs that test when lesion burden does or does not function as a severity proxy	Pathology load/distribution, synapse measure, electrophysiology or oscillation readout, connectivity proxy, behavior	Pathology burden is a defensible severity proxy only when circuit-level disruption and behavior are measured directly and move with the pathology claim.
Q2	Amyloid/tau background compared with the same background plus measured vascular stress or vascular rescue (e.g., hypoperfusion, BBB injury, CAA, SVD-like burden)	Direct vascular phenotyping, pathology, white-matter or connectivity readout, behavior	Vascular-modifier language is justified only when the vascular component is measured rather than assumed from worsened behavior alone.
Q3	Immune/glial manipulation designs that test temporality and necessity/sufficiency relative to pathology and circuit change	Immune/glial-state markers, timing relative to pathology, synapse/circuit readout, behavior	"Driver" claims require that the inflammatory or glial-state change precede the downstream phenotype and that altering the pathway changes the phenotype.
Q4	Multi-domain behavioral battery interpreted alongside explicit confound controls and at least one synapse or circuit readout	Memory assay, executive/attention assay, ADL proxy, motor, vision, and anxiety controls, circuit or synapse endpoint	Dementia-relevant impairment is best supported by convergent multi-domain evidence rather than by a single task.
Q5	Intervention studies with repeated measures and treatment-appropriate time-course checks rather than single-time-point rescue comparisons	Target engagement, circuit preservation, longitudinal slopes, nonlinear or segmented trajectory models when stage transitions are expected, washout or delayed retest only when pharmacologically relevant, behavior	Disease-modifying language is most defensible when target engagement, circuit preservation, and trajectory change align.

Table S2. Cross-cutting confounds, pitfalls, and endpoint-alignment cues

Domain	Frequent pitfall	Required alignment cue
Age and disease stage	Using very young animals to support late-life translational claims without clarifying that the study is mechanistic rather than syndrome-level	State whether age is a translational feature or a mechanistic simplification, and align claims accordingly.
Background strain and sex	Treating strain or sex differences as noise, then generalizing broadly from one background	Pre-specify whether heterogeneity is part of the design; if not, explicitly bound the inference to the tested background.
Behavioral interpretation	Calling a single-task improvement "cognitive rescue" despite motor, anxiety, or sensory confounds	Pair each behavioral endpoint with locomotor, vision/sensation, and anxiety/motivation controls.
Mixed-pathology claims	Invoking vascular or inflammatory contributions without measuring the claimed co-pathology	Add direct phenotype readouts for the second process, not just worse behavior or worse pathology.
Target engagement and clinical meaning	Treating biomarker change as equivalent to patient-relevant benefit	Require circuit preservation and/or longitudinal functional change before using disease-modification language.
Negative dissociations	Omitting biomarker–behavior discordance from the interpretation	Report the dissociation explicitly and interpret it as mechanistically informative rather than as a nuisance result.

Table S3. Operational decision rules for heterogeneity, age, and comorbidity

Scenario	Minimum design rule	Interpretive consequence if omitted
Late-life translation claim	Use aged animals, stage-matched intervention timing, or explicitly label the study as an early-mechanism model	Overstates relevance to late-life dementia and underestimates reserve, resilience, and mixed-pathology effects.
Heterogeneity included by design	Treat sex, age, or background as stratification variables with prespecified comparisons	Broad claims become difficult to interpret because variability is neither modeled nor clearly limited.
Comorbidity or mixed pathology claim	Measure the additional pathology invoked (vascular injury, inflammation, Lewy-like burden, etc.) in the same experiment	"Mixed pathology" becomes descriptive rhetoric rather than an evidence-based inference.
Reserve or resilience claim	Demonstrate preserved cognition/function despite documented pathology burden	Reserve/resilience language becomes indistinguishable from underpowered pathology-behavior correlation.
Mechanism-to-translation bridge	State whether heterogeneity is intended to improve construct validity or isolate a causal mechanism	The reader cannot tell whether simplification is a feature or a limitation of the design.

Table S4. Minimum behavioral battery and confound-control panel

Component	Preferred measure	Required control	Interpretation note
Memory domain	One pre-specified memory assay with replication or a conceptually related alternate format	Training exposure, exclusion rules, and performance criteria reported in advance	A single memory task should not carry the syndrome-level claim alone.
Executive/attention domain	One assay that samples flexibility, attention, or response control	Match task demands across groups and report motivation-related exclusions	Adds construct coverage beyond hippocampal memory alone.
ADL proxy	Nest building, burrowing, or another ethological functional readout	Housing and handling conditions reported consistently across groups	Improves face validity by sampling everyday-function analogs.
Home-cage or digital readout	Sleep–wake structure, circadian fragmentation, locomotor structure, or grooming profile	Stable recording conditions and transparent preprocessing	Helps capture low-stress, longitudinal functional change.
Motor, vision, and anxiety panel	Locomotion/strength, vision or sensation screen, and anxiety/motivation assay	Collected in the same animals used for the primary cognitive claim	If these move with the cognitive endpoint, the behavioral conclusion remains provisional.
Circuit support	At least one synapse or circuit endpoint paired with the battery	Same-stage sampling as the behavioral window	Strengthens the link between task performance and neural-system integrity.

Table S5. Search transparency and citation-update log

Component	Details	Purpose / selection rationale
Review type and scope	Conceptual narrative review with a structured literature-selection and citation-audit matrix; not a PRISMA systematic review or meta-analysis.	Preserves the question-first aim of the manuscript while making the citation base checkable.
Sources queried / verified	PubMed was used as the main biomedical/preclinical source; OpenAlex was used for broader scholarly coverage; Crossref was used for DOI and publisher-metadata verification; Semantic Scholar was queried as an additional academic graph source. Direct Scopus and Web of Science querying was not performed because institutional/API access was unavailable, and Google Scholar was not scraped.	Uses transparent, reproducible metadata sources and avoids implying coverage from databases that were not directly queried.
Search period and foundation sources	Targeted checks focused primarily on literature published from 2000 to March 2026; older seminal papers were retained when needed for clinicopathological correlation, synaptic/circuit biology, behavioral phenotyping, or staging logic.	Balances recent model/biomarker literature with older framework-setting or foundational studies.
Q–M–O search domains	Searches were organized around seven domains: Q1 pathology/proteinopathy/model validity, Q2 vascular dysfunction/mixed pathology, Q3 immune/glial state, Q4 behavioral construct validity, Q5 treatment/disease modification, biomarker/clinical framework, and model design/reproducibility.	Aligns source discovery with the five manuscript questions plus cross-cutting biomarker and design domains.
Core search concepts	Alzheimer disease, dementia, animal model, mouse model, transgenic, knock-in, amyloid, tau, vascular cognitive impairment, mixed pathology, neuroinflammation, synaptic loss, circuit dysfunction, behavioral assay, translation, preclinical, APOE, TREM2, sex, aging, biomarker, and AT(N).	Ensures that records can be mapped to model type, biological mechanism, endpoint level, and translational interpretation.

Table S5 continued.

Component	Details	Purpose / selection rationale
Raw search record count	The supplementary query log contains 29 Q–M–O-domain searches returning 1,344 raw records: PubMed = 920, OpenAlex = 259, Crossref = 165, Semantic Scholar = 0 retained query returns.	Reports an auditable search breadth without presenting the workflow as exhaustive systematic screening.
Deduplicated candidate screening	After DOI/title merging, core-reference overlap checks, and relevance screening, 1,044 deduplicated hybrid candidates were screened for fit to the review scope.	Gives a concrete screened denominator for the citation-audit matrix.
Final literature matrix	Supplementary Data 1 contains 320 DOI-verified records: 98 retained core records and 222 related unused records. The 98 core records consist of 97 journal articles and one book/reference record, all cited in the revised main text.	Distinguishes the submitted citation base from the broader verified candidate pool.
Inclusion logic	Records were retained or classified when they concerned AD/dementia-relevant mechanisms, animal-model validity, preclinical interpretation, translational endpoints, amyloid/tau, vascular dysfunction, immune/glial state, synapse/circuit biology, behavioral construct validity, biomarker mapping, treatment interpretation, or reproducibility/design logic.	Makes inclusion operational at the level of Q–M–O relevance rather than citation availability alone.
Exclusion or related-unused logic	Records were excluded from detailed synthesis or retained only as related unused records when they were duplicates, lacked DOI/PMID-backed metadata, lacked clear AD/dementia relevance, could not be mapped to a Q–M–O domain, were narrow method/assay papers without model–outcome interpretive value, or were redundant with stronger or more seminal sources already cited.	Explains why related but unused records were not all carried into the manuscript.
Selection-flow summary	1,344 raw query returns → 1,044 deduplicated hybrid candidates screened → 320 DOI-verified records in the audit matrix → 98 retained core references cited in the main text and 222 related unused records retained for transparency/literature expansion.	Provides a checkable flow summary without claiming PRISMA-level systematic coverage.

Note. These counts document a structured citation-audit trail for a conceptual review. The matrix should not be read as a PRISMA flow diagram or as a claim that all potentially relevant database hits were identified, deduplicated, screened, and excluded systematically. ChatGPT/ChatGPT Prism was used only as an AI-assisted organization and formatting aid rather than as an independent evidence source; the authors reviewed and verified citation-to-claim alignment.

Table S6. Primary-study examples: fulfilled and not fully fulfilled Q–M–O requirements

Question	Representative primary example	Requirement fulfilled / illustrated	Requirement not fully fulfilled / narrower interpretation
Q1	Busche et al. in vivo tau/amyloid circuit study (Busche et al. 2019)	Shows why lesion burden alone is an incomplete severity proxy by linking tau/amyloid context to circuit-level impairment.	Does not by itself provide a complete dementia-syndrome endpoint set; behavioral and longitudinal functional claims still require matched outcomes.
Q2	APP-overexpression plus chronic hypoperfusion study (Yamada et al. 2011)	Illustrates a proteinopathy + vascular-stress contrast and the need to measure both cognition and amyloid-related biology.	Broad hypoperfusion does not isolate a region-specific vascular pathway, and without vascular-rescue arms it supports overlapping-burden language better than independent-driver language.
Q3	NLRP3/caspase-1 perturbation in APP/PS1 mice (Heneka et al. 2013)	Provides a causal immune-pathway test by showing that inflammasome disruption changes pathology and cognitive outcomes.	Does not fully resolve stage specificity, multi-cell context, peripheral immune contribution, or mouse–human immune-state equivalence.
Q4	Touchscreen operant platform for rodent working memory/executive function (Oomen et al. 2013)	Illustrates how task design can broaden construct coverage beyond a single hippocampal maze assay.	A behavioral platform still needs disease-stage matching, sensory/motor/motivation controls, and synapse/circuit support before syndrome-level claims are made.
Q5	Engram-cell activation in early AD mouse models (Roy et al. 2016)	Shows that a behavioral rescue can identify a circuit mechanism and distinguish retrieval failure from global pathology reversal.	Supports a limited circuit-rescue interpretation; it should not be read as disease modification without target engagement, trajectory change, and broader functional endpoints.

Table S7. Companion Q–M–O Decision Tree Simulator: implementation summary

Simulator component	Implementation in the web tool	Interpretation / boundary
Access and code	The simulator was designed and developed by the authors as an optional companion implementation of the manuscript framework. Live tool: Q–M–O Decision Tree Simulator; source repository: GitHub repository. The local implementation is a browser-based HTML/React/Tailwind application.	Provided as an applied companion to the manuscript framework; it is not a new dataset, diagnostic device, prognostic instrument, or validated regulatory decision tool.
Decision inputs	Six user-facing steps: biological modifiers; target engagement; translation-aligned time course; synaptic/circuit preservation; longitudinal trajectory/slope change; and biomarker–behavior concordance.	Mirrors the Question 5 logic for bounding claims about "improvement" in animal models.
Scoring logic	The implementation assigns heuristic points to each step (maximum 100): modifiers 20, target engagement 20, time course 15, circuit preservation 20, trajectory change 15, and concordance 10. Partial credit is available for modifier control and structural-only circuit evidence.	The score is a structured confidence aid for manuscript interpretation; it should not be treated as an effect size, clinical endpoint, or substitute for pre-specified statistical analysis.
Branching claim labels	The tool gives bounded labels such as symptomatic/off-target effect, confined claim, acute/transient performance enhancement, mechanistic dissociation, basic target engagement, circuit-preserving/neuroprotective effect, or strong disease-modifying claim.	Labels are intended to reduce over-interpretation by matching the strongest permissible claim to the weakest missing evidence stream.
Optional AI-assisted drafting	The HTML implementation includes an optional Gemini API prompt that can draft a concise Discussion/Limitations paragraph from the user's selected answers, score, and claim boundary.	Any generated text is only a drafting aid; users must verify all scientific statements, limitations, citations, and claim boundaries before use. It is not an independent evidence source.

REFERENCES

Busche, M. A. et al. (2019). "Tau impairs neural circuits, dominating amyloid-β effects, in Alzheimer models in vivo". In: *Nature Neuroscience* 22.1, pp. 57–64. DOI: 10.1038/s41593-018-0289-8.

Heneka, M. T. et al. (2013). "NLRP3 is activated in Alzheimer's disease and contributes to pathology in APP/PS1 mice". In: *Nature* 493.7434, pp. 674–678. DOI: 10.1038/nature11729.

Oomen, C. A. et al. (2013). “The touchscreen operant platform for testing working memory and pattern separation in rats and mice”. In: *Nature Protocols* 8.10, pp. 2006–2021. DOI: 10.1038/nprot.2013.124.

Roy, D. S., Arons, A., Mitchell, T. I., Pignatelli, M., Ryan, T. J., and Tonegawa, S. (2016). “Memory retrieval by activating engram cells in mouse models of early Alzheimer’s disease”. In: *Nature* 531.7595, pp. 508–512. DOI: 10.1038/nature17172.

Yamada, M. et al. (2011). “The Influence of Chronic Cerebral Hypoperfusion on Cognitive Function and Amyloid β Metabolism in APP Overexpressing Mice”. In: *PLoS ONE* 6.1, e16567. DOI: 10.1371/journal.pone.0016567.
